# Systematic investigation of density functional theory methods for diorganotin 1,3-dithiole-2-thione-4,5-dithiolate complexes, R_2_Sn(dmit)

**DOI:** 10.1007/s00894-026-06990-3

**Published:** 2026-10-13

**Authors:** Raíssa Ribeiro Domingues Brum, Jonathan B. Brum, Glaucio Braga Ferreira

**Affiliations:** 1 Graduate Program in Chemistry, Chemistry Institute, Fluminense Federal University, Outeiro de S. João Batista s/n., Centro, Niterói, Rio de Janeiro 24210-130 Brazil; 2 Department of Inorganic Chemistry, Chemistry Institute, Fluminense Federal University, Outeiro de S. João Batista s/n., Centro, Niterói, Rio de Janeiro 24210-130 Brazil

**Keywords:** Systematic Study, Organotin complexes, Dmit ligand, DFT

## Abstract

**Context:**

Organotin complexes have been extensively studied due to their versatile structural features and potential applications in catalysis, materials science, and bioinorganic chemistry. Among them, compounds containing Sn–S interactions are of particular interest, as the tin–sulfur bond strongly affects both the stability and the electronic properties of the complexes. In addition, theoretical studies on 1,3-dithiole-2-thione-4,5-dithiolate systems have attracted considerable attention because of their potential technological applications.

**Methods:**

Density functional theory calculations were performed to evaluate the performance of different exchange–correlation functionals in describing the structural and spectroscopic properties of R_2_Sn(dmit) complexes. A total of 36 levels of theory were assessed for geometry optimization by combining 12 density functionals with three basis sets (def2-SVP, def2-TZVP, and def2-QZVP). The calculated geometric parameters were compared with experimental crystallographic data using statistical metrics including mean deviation, mean absolute deviation, standard deviation, and root mean square deviation. The results show that the choice of exchange–correlation functional has a greater influence on the accuracy of the optimized geometries than the basis-set size, with PBE/def2-SVP, BP86/def2-SVP, and M06-2X/def2-TZVP providing the best overall agreement with the experimental structures. Harmonic vibrational frequencies were corrected by linear scaling and compared with the experimental infrared spectra, demonstrating that BP86 and PBE consistently produced the lowest statistical deviations, whereas increasing the basis-set size beyond the triple-ζ level provided only marginal improvements. The ^119^Sn NMR chemical shifts were calculated using the ZORA, DKH, and X2C relativistic Hamiltonians. The double-hybrid B2PLYP-D3 functional yielded the lowest absolute errors overall within the investigated dataset. For most global and range-separated hybrid functionals (e.g., CAM-B3LYP, M06-2X, and ωB97X-D3), as well as the GGA functional BP86, the X2C Hamiltonian provided satisfactory agreement with the experimental data within the investigated dataset, yielding *R*^2^ values of 0.984-0.986, regression slopes moderately close to unity, and low mean absolute deviations. The chemical-shift range covered by the investigated systems represents only a subset of the full ^119^Sn chemical-shift range, reflecting the specific chemical space considered in this study. Overall, the results demonstrate that computationally efficient GGA functionals provide a favorable balance between accuracy and computational cost for geometry optimization and vibrational frequencies, whereas the prediction of ^119^Sn NMR chemical shifts is strongly influenced by the choice of relativistic treatment, B2PLYP-D3/ZORA being the top-performing individual protocol and X2C providing the most consistent agreement with the experimental data for most functionals for the systems investigated.

**Graphical Abstract:**

**Figure:**
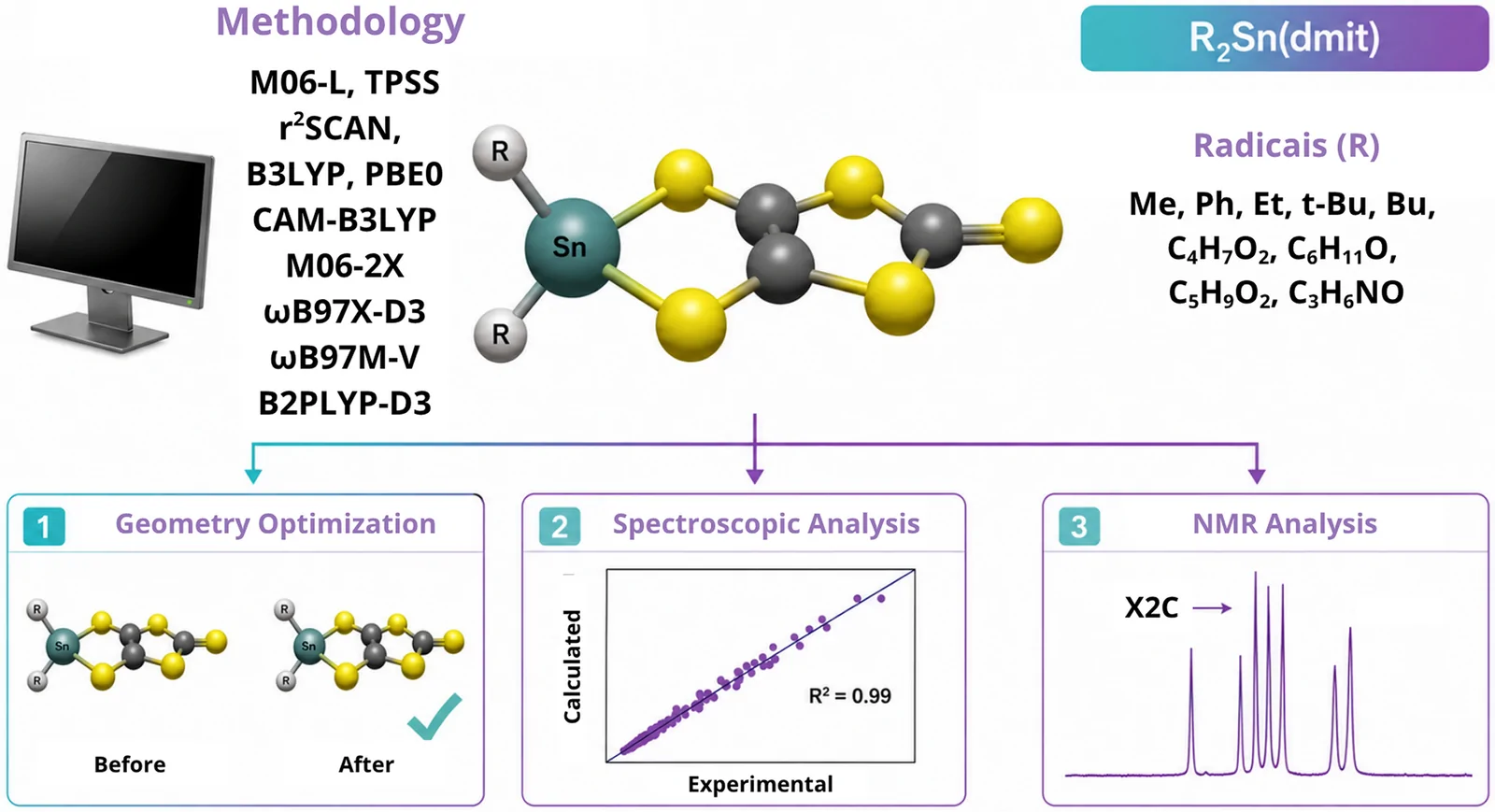

**Supplementary Information:**

The online version contains supplementary material available at https://doi.org/10.1007/s00894-026-06990-3.

## Introduction

Organotin complexes have attracted attention because of their diverse structural characteristics and wide range of applications [1–3]. These compounds exhibit versatile coordination chemistry, allowing the formation of stable structures with different coordination numbers and geometries, typically adopting octahedral or distorted trigonal bipyramidal arrangements [4, 5]. This structural flexibility is related to their adjustable electronic properties, making organotin(IV) compounds promising candidates for applications in catalysis, materials science, and electronic devices [6]. Their incorporation into hybrid materials has demonstrated potential in photovoltaic and semiconductor technologies, highlighting their relevance in advanced functional materials [7–9].

The Sn–S bond in tin complexes significantly influences their structural and electronic properties. The interaction between tin and sulfur atoms typically exhibits significant covalent character, strongly affecting the structural stability and reactivity of the complexes [10]. The Sn–S bond length and the coordination environment depend on factors such as the oxidation state and coordination number of the tin center, which can range from four to seven coordinate species [11]. These interactions are particularly important in thiolate and dithiolate systems, where sulfur atoms act as electron density donors, stabilizing the metal center and modulating its electrophilicity [12, 13]. Furthermore, Sn–S bonds play a crucial role in applications such as thin-film deposition, catalysis, and biological activity, reinforcing their importance in organotin chemistry [14].

**Fig. 1:**
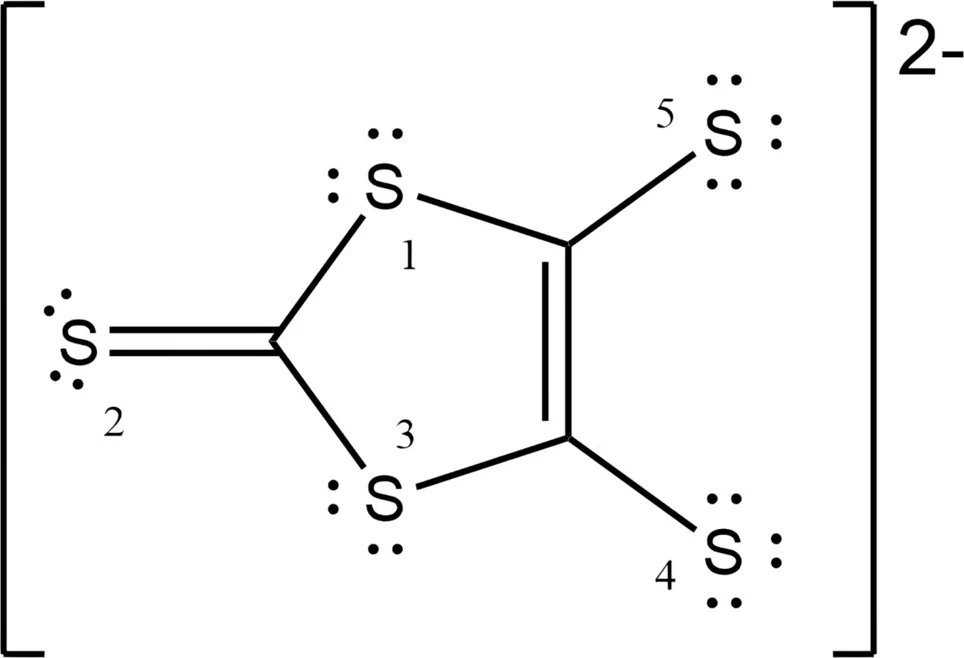
1,3-Dithiole-2-thione-4,5-dithiolate (dmit) structure

The 1,3-dithiole-2-thione-4,5-dithiolate (dmit) ligand (Fig. 1) is particularly relevant because of its rich electronic structure and versatile coordination chemistry. This arises from a soft–soft acid–base interaction, according to Pearson’s HSAB concept [15]. As a sulfur chelating ligand, dmit forms stable complexes with a wide variety of metals, exhibiting remarkable electronic, magnetic, and optical properties [16, 17]. Compounds containing the dmit ligand have been extensively studied because of their applications in molecular electronics, nonlinear optics, and conductive materials, as they may exhibit electrical conductivity, magnetism, and charge-transfer behavior [18, 19].

The combination of organotin units with the dmit ligand gives rise to systems that integrate the advantages of both components. These systems have attracted increasing interest due to their potential applications in optoelectronics, energy storage, catalysis, and sensing technologies [20]. The combination of organic substituents and inorganic coordination environments enables modulation of the structural and electronic properties of these systems [21].

Diorganotin(dmit) complexes, R_2_Sn(dmit), represent an interesting class of compounds that combine the structural flexibility of organotin units with the electronic accessibility of the dmit ligand. Experimental studies [22] have shown that these complexes typically adopt distorted trigonal bipyramidal geometries, with significant intermolecular interactions, such as Sn···S and S···S contacts, contributing to their supramolecular organization. Despite their promising properties and potential applications, theoretical and computational studies on these systems remain relatively limited, particularly with regard to a systematic evaluation of their structural and spectroscopic properties.

In this work, a systematic investigation was carried out to evaluate the performance of DFT methods in describing the structural and spectroscopic properties of R_2_Sn(dmit) complexes, including infrared spectra and ^119^Sn NMR chemical shifts, with the aim of identifying reliable computational protocols. These results are expected to contribute to a better understanding of the properties of these complexes and to support future studies on related organotin systems.

## Computational details

Geometry optimizations were performed in the gas phase using the ORCA program package (version 6.1.0) [23]. The initial structures were generated from CIF files obtained from the Cambridge Crystallographic Data Centre (CCDC) [24]. To provide a representative assessment across different rungs of Jacob’s ladder, 12 density functionals spanning distinct exchange–correlation approximations were selected: the GGA functionals PBE [25, 26] and BP86 [27, 28]; the meta-GGA functionals M06-L [29], TPSS [30], and r^2^SCAN [31, 32]; the global hybrid functionals B3LYP [33–35], and PBE0 [36]; the global hybrid meta-GGA functional M06-2X [37]; the range-separated hybrid functionals CAM-B3LYP [38], and ωB97X-D3 [39, 40]; the range-separated hybrid meta-GGA functional ωB97M-V [41]; and the double-hybrid functional B2PLYP-D3 [42]. These functionals were combined with the def2-SVP, def2-TZVP, and def2-QZVP basis sets [43, 44], representing split-valence, triple-ζ, and quadruple-ζ qualities, respectively. This basis-set hierarchy was selected to systematically evaluate basis-set convergence while maintaining consistency within the well-established def2 family, which has been employed for main-group and heavy-element compounds, including organotin systems, in previous studies [45–47]. Normal mode calculations were carried out at the same level of theory to confirm the nature of the stationary points as true minima on the potential energy surface and to compare the theoretical results with available experimental infrared data.

A conformer-rotamer ensemble (CRE) was generated for each compound with CREST 3.0.2 [48] using GFN-FF [49] or GFN2-xTB [50]. The conformer–rotamer ensemble was further refined using CENSO 2.1.1 [51], yielding the corresponding Gibbs free energies and Boltzmann populations. Geometry optimizations were performed at the r^2^SCAN-3c/def2-TZVP level of theory, which is commonly employed as a reference level for this type of conformational analysis [47], in conjunction with the SMD implicit solvation model to account for solvation effects in acetone. Boltzmann populations were determined using the modified rigid-rotor harmonic-oscillator (mRRHO) approach. The incorporation of Boltzmann weighting resulted in an average absolute variation of approximately 0.75 ppm in the MAD values relative to those obtained using the lowest-energy conformer. The corresponding statistical comparison is provided in Table S1 of the Supporting Information.

NMR shielding constants were calculated for the lowest-energy conformer using the Gauge-Including Atomic Orbital (GIAO) method [52–55], as implemented in ORCA 6.1.0. Since electrons close to the nucleus of heavy elements, such as tin, are significantly influenced by relativistic effects, the inclusion of scalar relativistic corrections is essential for an accurate description of NMR properties [47]. Scalar relativistic effects were treated using three distinct formalisms: the Zero-Order Regular Approximation (ZORA) Hamiltonian [56–58], which approximates relativistic effects through a regular expansion of the Dirac equation and is computationally efficient for heavy-element systems; the Exact Two-Component (X2C) method [59–63], which achieves exact decoupling of the one-electron Dirac Hamiltonian; and the Douglas–Kroll–Hess (DKH2) Hamiltonian to second order [64–66], which decouples the Dirac equation through a sequence of unitary transformations expanded in the external potential. These approaches were selected because they are widely used relativistic formalisms for quantum-chemical calculations involving heavy elements and provide different compromises between computational cost and accuracy [56, 67, 68]. For DKH2, DKH-def2-TZVP was used for the non-Sn atoms and SARC-DKH-TZVP for Sn; for ZORA, ZORA-def2-TZVP was used for the non-Sn atoms and SARC-ZORA-TZVP for Sn; and X2C-TZVPall-s was employed for the X2C calculations. All relativistic ^119^Sn NMR calculations were performed using an all-electron treatment of the Sn atom; no effective core potential was employed for Sn. The relativistic NMR calculations employed the full picture-change treatment implemented in ORCA 6.1.0 for core-sensitive properties such as NMR, following the ORCA 6.1 documentation. For the X2C/GIAO calculations, restricted magnetic balance (RMB) was employed for the small-component basis functions.

Their inclusion enables a systematic assessment of the influence of the relativistic Hamiltonian on the calculated ^119^Sn NMR properties, allowing the identification of the approach that provides the best agreement with the available experimental data. The NMR data reported in the literature are typically collected in solution [69–71], so solvent effects were taken into account using the solvation model based on density (SMD) [72]. For the ^119^Sn NMR shielding calculations, the SMD solvent was selected to match the experimental solvent reported for each compound (Table S7).

### General considerations

**Fig. 2:**
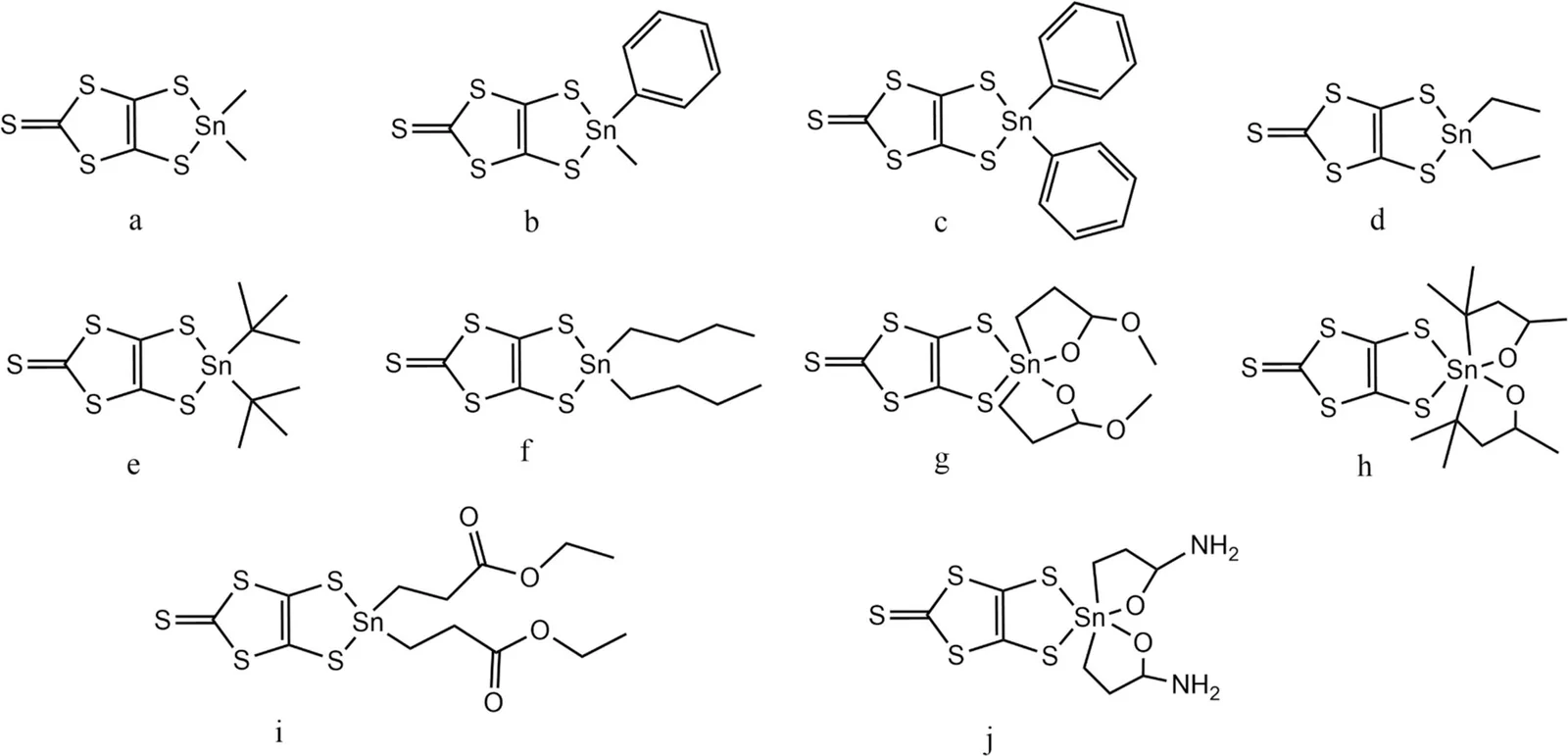
Geometries of diorganotin 1,3-dithiole-2-thione-4,5-dithiolate compounds, R_2_Sn(dmit) complexes. **a** Me_2_Sn(dmit) [71], **b** MePhSn(dmit) [22], **c** Ph_2_Sn(dmit) [78], **d** Et_2_Sn(dmit) [71], **e** t-Bu_2_Sn(dmit) [74], **f** Bu_2_Sn(dmit) [22], **g** (C_4_H_7_O_2_)_2_Sn(dmit) [73], **h** (C_6_H_11_O)_2_Sn(dmit) [75], **i** (C_5_H_9_O_2_)_2_Sn(dmit) [77], **j** (C_3_H_6_NO)_2_Sn(dmit) [76]

The structural analysis used Sn–S bond lengths, as well as the R–Sn–R bond angle, as reference parameters. The vibrational analysis was performed using the normal modes calculated for structure *e* (Fig. 2). The ^119^Sn nucleus was selected for the NMR chemical-shift analysis. The results were analyzed through comparison with experimental data [22, 73–77]. Tetramethyltin (SnMe_4_) was used as the reference [47] compound for calculating the ^119^Sn NMR chemical shifts according to Eq. 1

1 $$\delta {(}^{119}Sn)=\sigma {(}^{119}{Sn}_{Sn{Me}_{4}})-\sigma {(}^{119}Sn)$$

### Dataset

The structural parameters were compared with the available crystallographic data (Fig. 2) [22, 71, 73–78]. Each complex contains a single tin atom as the metal center, one 1,3-dithiole-2-thione-4,5-dithiolate (dmit) ligand, and two organic ligands (R), in which the R groups may be mono- or bidentate (R and R’ = Me, Ph, Et, t-Bu, Bu, C_4_H_7_O_2_, C_6_H_11_O, C_5_H_9_O_2_, C_3_H_6_NO). The first stage of this study involved optimizing the geometries of the selected complexes to evaluate the performance of the different levels of theory tested in this systematic study.

The butyl derivative was excluded from the geometrical parameter analysis due to the absence of experimental crystallographic data. Experimental ^119^Sn NMR chemical shifts were available for seven of the ten R₂Sn(dmit) complexes. Four additional Sn–S complexes were therefore included exclusively in the NMR benchmark, resulting in a total NMR dataset of 11 compounds. These compounds were selected to increase both the number of experimental data points and the range of ^119^Sn chemical shifts covered by the regression analysis, providing a more robust statistical evaluation of the computational protocols while preserving the systematic investigation of the organotin-dmit complexes as the primary focus of the study. The additional compounds exhibit structural and electronic features comparable to those of the organotin-dmit complexes investigated in this work, including comparable coordination numbers, charge states, and Sn–S coordination environments. The molecular structures of the four additional compounds included in the NMR analysis are shown in Figure S1 (Supporting Information).

### Statistical analysis

The geometric parameters (Sn–S bond lengths and the R–Sn–R bond angle) were normalized using the min–max scaling method to place all values on the same scale. The normalized values were obtained according to Eq. 2 [79].

2 $${x}_{\mathrm{n}\mathrm{o}\mathrm{r}\mathrm{m}}= \frac{x- {x}_{\mathrm{m}\mathrm{i}\mathrm{n}}}{{x}_{\mathrm{m}\mathrm{a}\mathrm{x}}-{x}_{\mathrm{m}\mathrm{i}\mathrm{n}}}$$

where x is the original value, and ${x}_{\mathrm{m}\mathrm{i}\mathrm{n}}$ and ${x}_{\mathrm{m}\mathrm{a}\mathrm{x}}$ correspond to the minimum and maximum values of the dataset, respectively.

The mean deviation (MD), mean absolute deviation (MAD), standard deviation (SD), root mean square deviation (RMSD), and coefficient of determination (*R*^2^) were calculated [47]. These statistical measures provide a quantitative assessment of the accuracy of the different computational methods used in this study.

The scaling corrections of the calculated vibrational frequencies were applied via linear regression (Eq. 3), where α and β represent the slope and intercept of the linear best fit between calculated and experimental data [47, 80].

3 $${x}_{\mathrm{s}\mathrm{c}\mathrm{a}\mathrm{l}\mathrm{e}\mathrm{d}}= \frac{{x}_{\mathrm{c}\mathrm{a}\mathrm{l}\mathrm{c}.}-\beta }{\alpha }$$

For the ^119^Sn NMR chemical shifts, the correlation between the calculated and experimental values was evaluated through linear regression analysis. The coefficient of determination (*R*^2^) was used to assess the proportion of variance in the experimental data explained by the theoretical model. The 95% confidence intervals for the regression parameters (slope and intercept) were calculated using the standard error of the estimates, and the *p*-value was obtained from the t-distribution with N-2 degrees of freedom to test the null hypothesis that the slope is equal to zero. These statistical measures provide a quantitative assessment of the correlation between the calculated and experimental ^119^Sn NMR chemical shifts, as well as the uncertainty associated with the regression parameters.

## Results

### Geometrical optimization

**Fig. 3:**
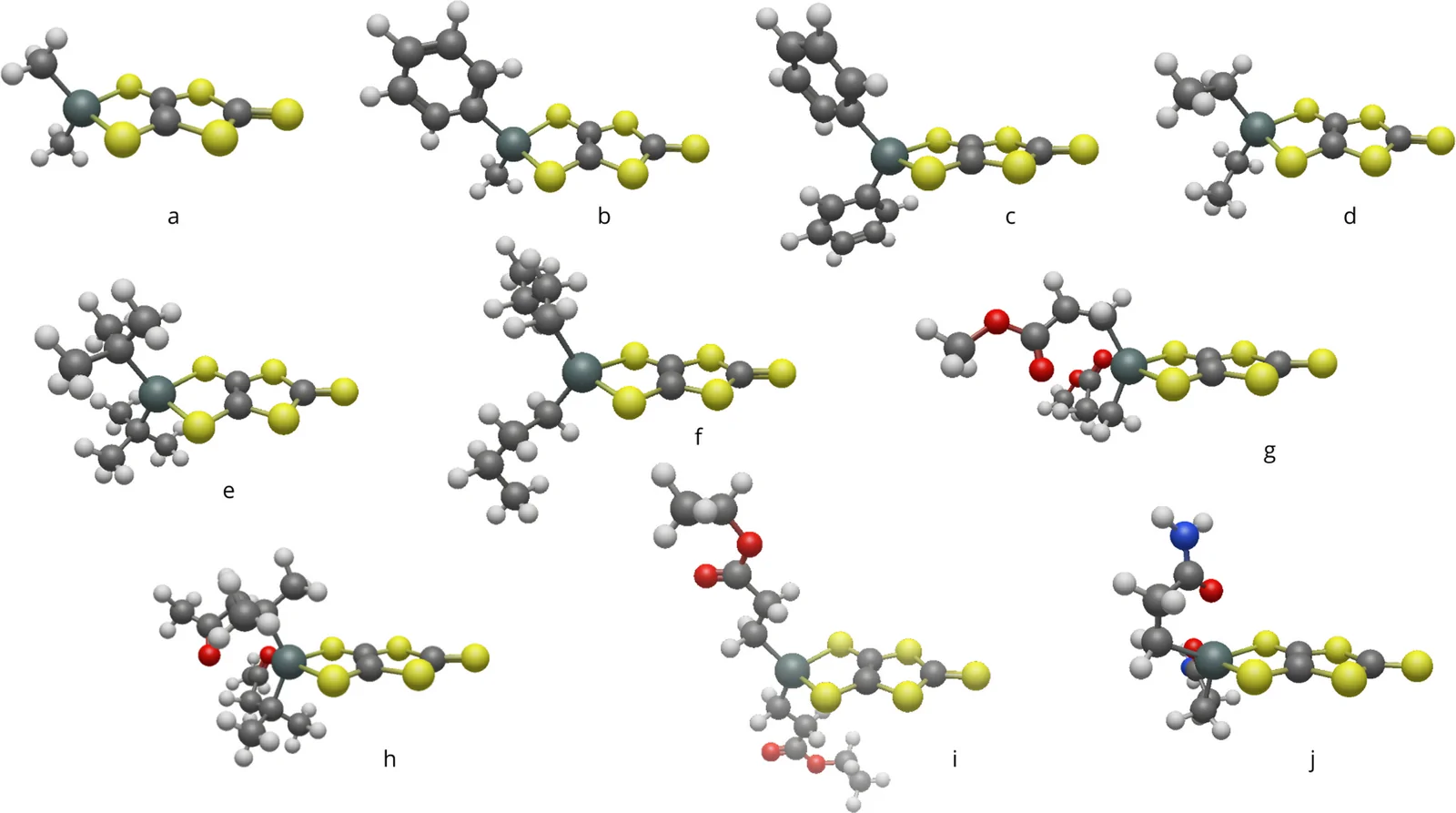
Optimized geometries of diorganotin 1,3-dithiole-2-thione-4,5-dithiolate compounds, R_2_Sn(dmit) complexes. **a** Me_2_Sn(dmit), **b** MePhSn(dmit), **c** Ph_2_Sn(dmit), **d** Et_2_Sn(dmit), **e** t-Bu_2_Sn(dmit), **f** Bu_2_Sn(dmit), **g** (C_4_H_7_O_2_)_2_Sn(dmit), **h** (C_6_H_11_O)_2_Sn(dmit), **i** (C_5_H_9_O_2_)_2_Sn(dmit), **j** (C_3_H_6_NO)_2_Sn(dmit)

The optimized geometries of the complexes are shown in Fig. 3, indicating that the main structural features are preserved, including the coordination environment of the tin center, the orientation of the dmit ligand, and the arrangement of the organic substituents. The tin center exhibits a coordination environment consistent with a distorted trigonal bipyramidal geometry, as commonly reported for diorganotin(dmit) derivatives containing non-coordinating R groups [22, 71, 74, 78].

From the perspective of molecular symmetry, when the R substituents are arranged symmetrically, these systems could be described as having C_2v_ or even D_3h_ symmetry; however, the steric and electronic effects imposed by the dmit ligand and the substituents lead to significant distortions, reducing the effective molecular symmetry [71, 81].

These results are consistent with the crystallographic data reported for R_2_Sn(dmit) complexes in the solid state, in which the tin center exhibits predominantly a five-coordinate tin center with a distorted trigonal–bipyramidal geometry. In these structures, one axial site is often occupied by a sulfur atom from the dmit ligand, while the second axial site is often associated with intermolecular Sn···S(thione) interactions, leading to supramolecular organization [71, 78]. Many derivatives, such as Me_2_Sn(dmit), Et_2_Sn(dmit), Ph_2_Sn(dmit), and t-Bu_2_Sn(dmit), form infinite one-dimensional chains or zigzag arrangements in the crystal lattice driven by these secondary Sn···S contacts [22, 71, 74, 78]. The nature and extent of these intermolecular interactions are influenced by the steric hindrance of the R groups, with bulkier substituents limiting intermolecular contacts and favoring arrangements with weaker interactions [74].

With regard to crystal symmetry, data from the literature indicate that R_2_Sn(dmit) complexes crystallize in various space groups, reflecting the delicate balance between molecular geometry, intermolecular interactions, and packing effects. Typically, these systems adopt orthorhombic or monoclinic crystal systems, with space groups such as *Pccn* [71], *Pna2*_*1*_ [71, 78], *Pcab* [22], and *P2*_*1*_*/c* [74, 76] frequently reported. Some derivatives, such as Me_2_Sn(dmit), crystallize in centrosymmetric space groups, while others, such as Et_2_Sn(dmit) and Ph_2_Sn(dmit), may adopt non-centrosymmetric and even polar space groups (e.g., *Pna2*_*1*_), leading to the directional alignment of molecules in the crystal lattice [71].

On the other hand, systems containing additional donor groups capable of coordinating to the metal center, such as ester or amide functional groups, tend to exhibit hexacoordinated environments with distorted octahedral geometries [73, 75–77]. In general, the optimized geometries obtained in this work are consistent with these structural trends reported in the literature, reinforcing the reliability of the theoretical approach in describing the structural characteristics of organotin(dmit) systems.

**Fig. 4:**
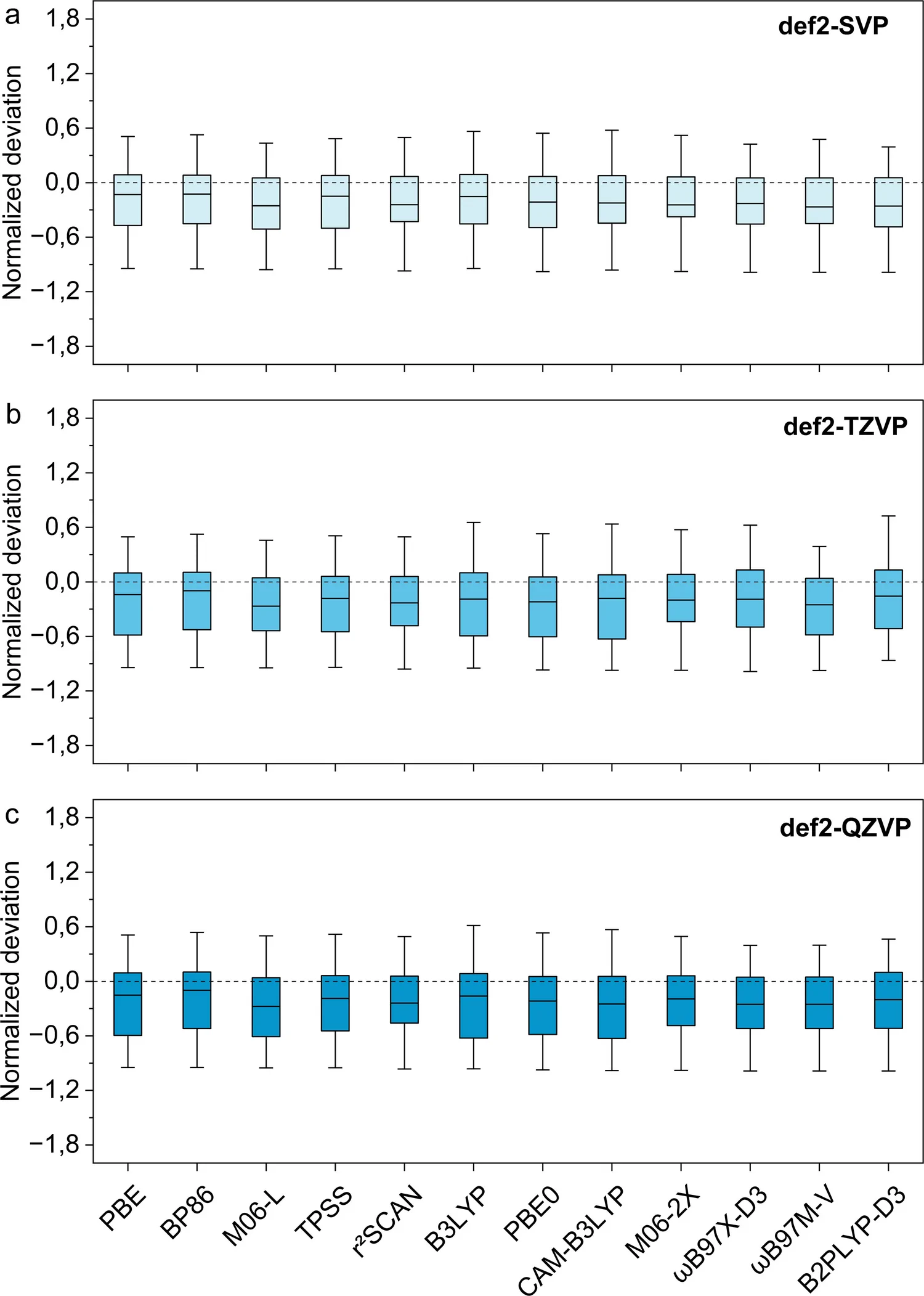
Box plots showing the deviations between calculated and experimental geometric parameters including Sn–S_1_ and Sn–S_2_ bond lengths and the R–Sn–R’ bond angle, for the 36 evaluated levels of theory. **a** def2-SVP; **b** def2-TZVP; **c** def2-QZVP

Figure 4 presents a quantitative comparison between calculated and experimental structural parameters [22, 73–77] in the form of box plots, illustrating the distribution of the deviations for the evaluated levels of theory. The experimental structural parameters are present in Table S2.

The calculated geometrical parameters were evaluated through comparison with the available crystallographic data, considering Sn–S bond lengths and R–Sn–R′ bond angles as representative structural descriptors of the investigated organotin complexes (Tables S3 and S4). Deviations between calculated and experimental values were observed for both bond distances and angular parameters, reflecting the differences between the isolated-molecule computational model and the solid-state experimental structures.

The statistical descriptors are reported in Table 1, including mean deviation (MD), mean absolute deviation (MAD), standard deviation (SD), and root mean square deviation (RMSD) obtained for the 36 evaluated levels of theory. Lower MAD and RMSD values indicate improved agreement between the computational predictions and experimental structural parameters, whereas MD values provide information regarding systematic overestimation or underestimation trends.

**Table 1:** Statistical parameters obtained for the optimized geometries of the R_2_Sn(dmit) complexes calculated at different DFT levels of theory, including mean deviation (MD), mean absolute deviation (MAD), standard deviation (SD), and root mean square deviation (RMSD)

	def2-SVP				def2-TZVP				def2-QZVP			
Functional	MD	MAD	RMSD	SD	MD	MAD	RMSD	SD	MD	MAD	RMSD	SD
B2PLYP-D3	− 0.267	0.352	0.453	0.370	− 0.173	0.366	0.444	0.425	− 0.221	0.359	0.454	0.404
B3LYP	− 0.191	0.338	0.432	0.392	− 0.217	0.389	0.478	0.432	− 0.217	0.384	0.476	0.430
BP86	− 0.196	0.329	0.427	0.384	− 0.200	0.343	0.440	0.397	− 0.199	0.344	0.441	0.398
CAM-B3LYP	− 0.212	0.348	0.444	0.395	− 0.228	0.384	0.481	0.429	− 0.236	0.378	0.475	0.418
M06-2X	− 0.224	0.339	0.435	0.377	− 0.219	0.336	0.430	0.375	− 0.239	0.347	0.446	0.382
M06-L	− 0.270	0.360	0.459	0.375	− 0.269	0.361	0.459	0.377	− 0.277	0.371	0.471	0.385
PBE	− 0.202	0.325	0.426	0.379	− 0.223	0.343	0.445	0.390	− 0.227	0.346	0.448	0.392
PBE0	− 0.224	0.349	0.447	0.392	− 0.245	0.357	0.459	0.394	− 0.245	0.357	0.458	0.393
r^2^SCAN	− 0.239	0.348	0.442	0.376	− 0.256	0.356	0.453	0.379	− 0.256	0.356	0.453	0.378
TPSS	− 0.223	0.333	0.434	0.376	− 0.229	0.343	0.443	0.384	− 0.231	0.345	0.445	0.385
ωB97M-V	− 0.248	0.350	0.445	0.374	− 0.273	0.358	0.465	0.382	− 0.271	0.349	0.453	0.369
ωB97X-D3	− 0.248	0.344	0.444	0.373	− 0.206	0.367	0.457	0.415	− 0.271	0.348	0.453	0.368

For the def2-SVP basis set, the evaluated functionals showed similar performances, with RMSD values ranging from 0.426 to 0.459. Among all methods, PBE provided the lowest RMSD value (0.426), followed closely by BP86 (0.427) and B3LYP (0.432). These results indicate that GGA functionals provide a reliable description of the investigated geometrical parameters when combined with the def2-SVP basis set. However, the relatively small differences among the best-performing methods suggest that functional selection has a moderate influence on the overall structural agreement within this computational level.

Although B3LYP did not provide the lowest RMSD, it exhibited the lowest systematic deviation among the evaluated functionals (MD = − 0.191), indicating reduced bias toward underestimation of the experimental reference values. In contrast, M06-L and B2PLYP-D3 showed the largest deviations within the def2-SVP series, with RMSD values of 0.459 and 0.453 and MAD values of 0.360 and 0.352, respectively. Notably, all evaluated functionals presented negative MD values, demonstrating a consistent tendency toward underestimation of the experimental geometrical parameters.

The TPSS functional exhibited competitive performance with an RMSD value of 0.434, while the range-separated hybrid functionals CAM-B3LYP and ωB97X-D3 showed intermediate results, both yielding RMSD values of 0.444. Similarly, ωB97M-V and PBE0 resulted in RMSD values of 0.445 and 0.447, respectively. These observations indicate that the inclusion of long-range exchange or dispersion corrections does not necessarily improve the reproduction of the experimental geometrical parameters for the present organotin complexes.

The use of the larger def2-TZVP basis set resulted in a modification of the functional ranking. M06-2X exhibited the lowest RMSD value among the evaluated protocols (0.430), representing the best-performing hybrid functional within this basis set. BP86 remained competitive (RMSD = 0.440), followed by TPSS (0.443). The B2PLYP-D3 functional also showed improved agreement compared with def2-SVP, decreasing its RMSD from 0.453 to 0.444, indicating that the effect of basis set enlargement is dependent on the functional employed.

In contrast, B3LYP and CAM-B3LYP displayed higher deviations with def2-TZVP, with RMSD values of 0.478 and 0.481, respectively. This behavior suggests that the balance between functional and basis-set errors influences the final performance of the computational protocol. The remaining functionals showed relatively small variations compared with the def2-SVP results.

For the def2-QZVP basis set, the overall performance pattern remained comparable to that observed for def2-TZVP. BP86 provided the lowest RMSD value (0.441), followed by TPSS (0.445) and M06-2X (0.446). PBE also maintained competitive performance, with an RMSD value of 0.448. The range-separated hybrid functionals ωB97M-V and ωB97X-D3 showed slight improvements compared with the def2-TZVP results, both yielding RMSD values of 0.453. Conversely, B3LYP and CAM-B3LYP continued to exhibit relatively larger deviations, with RMSD values of 0.476 and 0.475, respectively. More detailed information can be found in Table S5 in the SI.

The similar performance obtained with def2-TZVP and def2-QZVP suggests that increasing the basis set beyond the triple-ζ level provides limited benefits for the present dataset. Although larger basis sets generally allow a more complete description of the electron density, the remaining deviations appear to be dominated by other factors, including the intrinsic characteristics of the density functional approximation and differences between isolated-molecule theoretical structures and solid-state experimental geometries.

Similar to the def2-SVP results, all methods with def2-TZVP and def2-QZVP displayed negative MD values, indicating that increasing the basis set size does not eliminate the systematic underestimation observed for the investigated geometrical parameters. On average, 81.4%, 75.2%, and 76.6% of the calculated parameters were lower than the corresponding experimental values for def2-SVP, def2-TZVP, and def2-QZVP, respectively (Table S2). The decrease from 81.4% for def2-SVP to 75.2% for def2-TZVP suggests that basis set enlargement partially reduces this tendency; however, no additional improvement was observed at the QZVP level.

The systematic underestimation observed for the calculated geometrical parameters should be interpreted considering the differences between the theoretical and experimental models. The optimized structures correspond to isolated molecules in the gas phase, whereas the experimental reference values were obtained from single-crystal X-ray diffraction measurements, where crystal packing, intermolecular interactions, and temperature effects influence the observed molecular geometry.

Therefore, part of the deviations between calculated and experimental structures may arise from environmental effects rather than exclusively from limitations of the density functional approximation. Despite these differences, comparisons between gas-phase DFT geometries and crystallographic structures remain widely employed for organotin compounds, since experimental solid-state structures often represent the most reliable structural references available [18, 19, 81].

Because the same experimental dataset was consistently used for all computational protocols, the relative trends observed among the evaluated methods provide a meaningful basis for comparing their performance, even though absolute deviations may be affected by differences between the computational and experimental environments.

From a computational efficiency perspective, the def2-SVP basis set provides an attractive compromise between accuracy and computational cost for geometry optimization of the investigated organotin complexes. Nevertheless, the suitability of a given basis set remains dependent on the property under investigation, and the present conclusion is restricted to the geometrical parameters evaluated in this study.

Considering the combined performance of functional and basis set, the lowest RMSD values were obtained for PBE/def2-SVP (0.426), BP86/def2-SVP (0.427), and M06-2X/def2-TZVP (0.430). The favorable performance of the GGA-based protocols indicates that, for the structural descriptors considered here, relatively simple GGA functionals can achieve accuracy comparable to that of more computationally demanding approaches.

M06-2X/def2-TZVP represents the most accurate hybrid functional protocol among those evaluated in this study. Its performance shows that hybrid meta-GGA functionals can provide accurate structural predictions for organometallic and heavy-element-containing systems.

The B3LYP/def2-SVP protocol also exhibited competitive performance, with an RMSD value of 0.432 and the lowest systematic deviation among all evaluated combinations (MD = − 0.191). The well-established performance of B3LYP for molecular geometries, including organometallic compounds and systems containing heavier elements, is generally attributed to its balanced incorporation of exact exchange and correlation contributions. In the present study, this balance appears to reduce systematic bias, making B3LYP/def2-SVP an attractive alternative when minimizing systematic deviations is prioritized.

### Spectroscopic analysis

The vibrational properties of the R_2_Sn(dmit) complexes were evaluated through a comparison between calculated and experimental infrared frequencies. The complex (t-Bu)_2_Sn(dmit) was used as the reference system because it provides the most complete set of experimental IR data available in the literature [74]. The vibrational modes associated with the Sn–S, C–S, C = S, C = C, and C–H bonds were considered, as these vibrations represent the main structural features of the dmit ligand and the organotin coordination environment.

The calculated harmonic vibrational frequencies were evaluated against the experimental values after applying linear scaling corrections obtained from least-squares regression. This procedure was adopted to account for the systematic overestimation inherent to the harmonic approximation and to minimize method-dependent systematic errors before the statistical comparison. Consequently, the remaining deviations primarily reflect the ability of each density functional to reproduce the curvature of the potential energy surface rather than the intrinsic limitations of the harmonic approximation itself. The statistical parameters obtained after the scaling procedure are summarized in Table 2. The experimental vibrational frequencies together with the calculated values after linear scaling corrections are present in Table S6.

**Table 2:** Statistical parameters for the linearly scaled infrared (IR) vibrational frequencies (cm^−1^) for the R_2_Sn(dmit) complexes calculated at different DFT levels of theory, including mean deviation (MD), mean absolute deviation (MAD), and root mean square deviation (RMSD)

	def2-SVP			def2-TZVP			def2-QZVP*		
Functional	MD	MAD	RMSD	MD	MAD	RMSD	MD	MAD	RMSD
B2PLYP-D3	118.01	118.01	146.65	133.23	133.23	167.02	-	-	-
B3LYP	102.96	102.96	138.1	82.07	82.4	121.11	79.04	79.61	117.16
BP86	36.32	48.83	62.08	18.02	37.73	50.7	13.58	34.04	46.23
CAM-B3LYP	176.14	176.14	205.59	121.13	150.37	185.55	149.49	149.49	179.17
M06-2X	170.81	170.81	201.32	151.68	151.68	183.67	144.8	144.8	176.85
M06-L	118.47	118.47	146.98	99.43	99.43	137.03	113.97	113.97	168.71
PBE	21.42	37.02	51.76	30.98	41.48	56.38	26.62	37.48	51.1
PBE0	161.5	161.5	188.24	140.08	140.08	165.35	136.51	136.51	160.98
r^2^SCAN	107.68	107.68	134.78	87.76	87.76	115.12	84.68	84.68	110.08
TPSS	81.05	82.72	113.39	59.59	63.41	82.58	40.46	52.76	76.7
ωB97M-V	177.6	177.6	204.44	180.97	180.97	198.21	155.53	155.53	182.47
ωB97X-D3	195.61	195.61	219.91	173.95	173.95	199.68	155.83	155.83	182.07

Calculations at the B2PLYP-D3/def2-QZVP level were not performed

Overall, the correction substantially reduced the systematic deviations, allowing a meaningful comparison of the performance of the different exchange–correlation functionals and basis sets. Among all evaluated protocols, the GGA functionals consistently produced the smallest deviations. In particular, BP86 exhibited the best overall performance, with RMSD values of 62.08, 50.70, and 46.23 cm⁻^1^ for the def2-SVP, def2-TZVP, and def2-QZVP basis sets, respectively. Similarly, PBE provided highly competitive results, with RMSD values of 51.76, 56.38, and 51.10 cm⁻^1^ for the three basis sets. These results demonstrate that relatively simple GGA functionals are capable of accurately reproducing the vibrational spectra of the investigated organotin complexes after correction of the systematic harmonic error.

Among the hybrid functionals, B3LYP showed the best overall performance. Although its deviations remained larger than those obtained with BP86 and PBE, the RMSD decreased from 138.10 cm⁻^1^ with def2-SVP to 121.11 and 117.16 cm⁻^1^ with the def2-TZVP and def2-QZVP basis sets, respectively, indicating that this functional benefits from basis-set expansion. TPSS also showed a noticeable improvement as the basis set increased, with the RMSD decreasing from 113.39 cm⁻^1^ for def2-SVP to 82.58 cm⁻^1^ for def2-TZVP and 76.70 cm⁻^1^ for def2-QZVP.

**Fig. 5:**
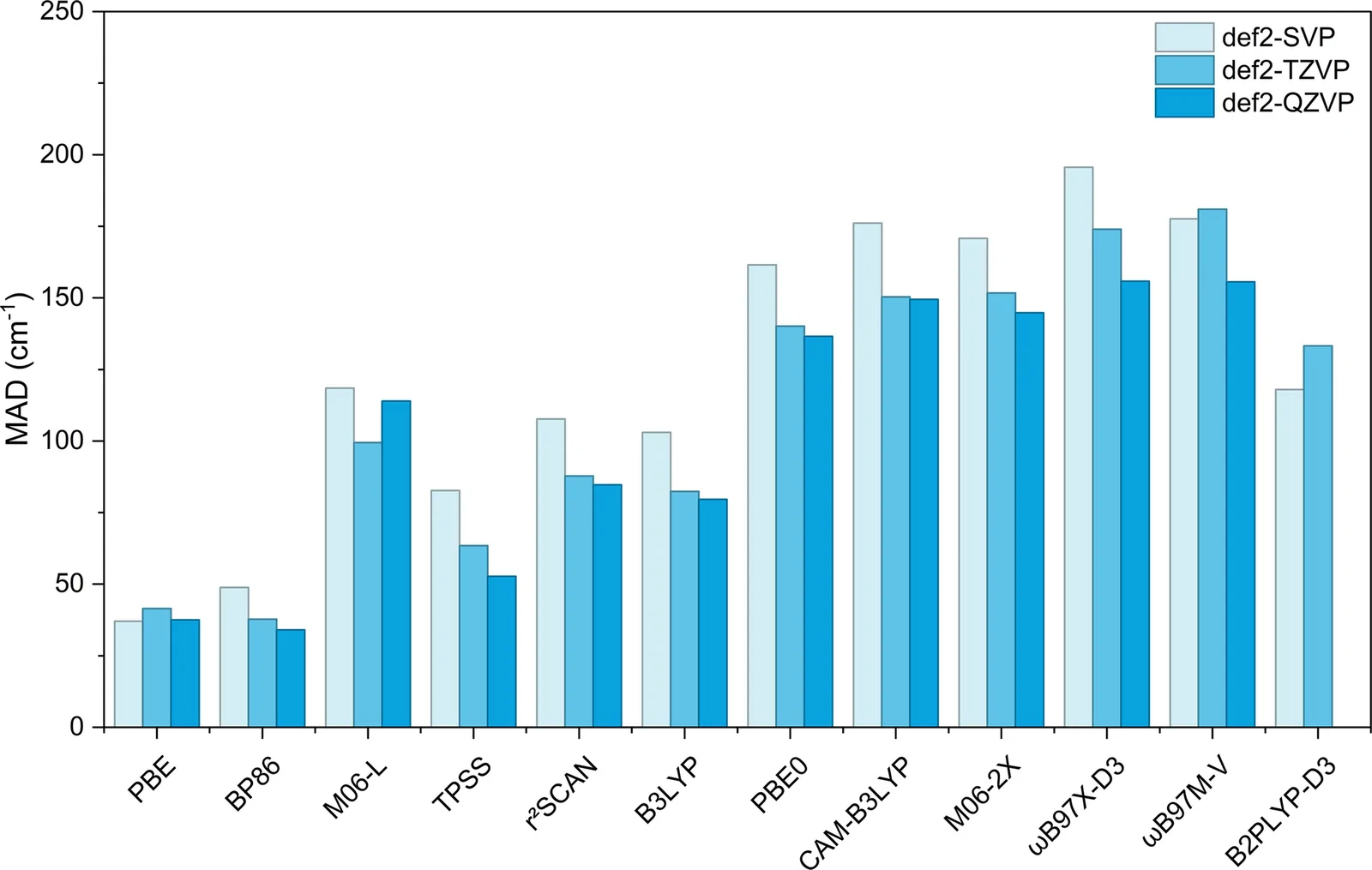
Variation of the MAD as a function of the density functional for the def2-SVP, def2-TZVP, and def2-QZVP basis sets

The influence of the basis set was comparatively small. For most functionals, the transition from def2-SVP to def2-TZVP produced only modest improvements, while the subsequent expansion to def2-QZVP resulted in only marginal changes (Fig. 5). These observations closely mirror those obtained in the structural study, where basis-set convergence was essentially achieved at the triple-ζ level. Therefore, the present results indicate that increasing the basis set beyond def2-TZVP provides limited benefits for the prediction of vibrational frequencies, while substantially increasing the computational cost.

The vibrational frequency calculations at the B2PLYP-D3/def2-QZVP level were not performed because the computational requirements of this protocol exceeded the available computational resources. Nevertheless, this omission is not expected to influence the conclusions of the present study. The structural analysis demonstrated that the expansion from def2-TZVP to def2-QZVP produced only marginal improvements, and an analogous trend was observed for the vibrational frequencies, where the quadruple-ζ basis set did not systematically improve the statistical descriptors obtained with the remaining functionals. Consequently, the absence of the B2PLYP-D3/def2-QZVP results does not compromise the assessment of basis-set effects or the comparison among the computational protocols investigated.

Overall, the results demonstrate that the choice of exchange–correlation functional exerts a considerably greater influence on the calculated vibrational frequencies than the basis-set size. BP86 and PBE consistently provided the most accurate predictions across the evaluated basis sets, whereas larger basis sets and more sophisticated hybrid or range-separated functionals did not yield systematic improvements. These findings reinforce the conclusions obtained from the structural analysis and indicate that computationally efficient GGA functionals combined with triple-ζ basis sets represent a favorable compromise between computational cost and predictive accuracy for the vibrational characterization of organotin complexes.

### NMR analysis

The ^119^Sn NMR chemical shifts were calculated to provide an independent evaluation of the computational methods investigated in this work (Tables S8-S10). NMR parameters are highly sensitive to the electronic environment surrounding the nucleus and therefore provide an additional assessment of the accuracy of the computational methods.

The use of effective core potentials (ECPs), such as those employed in the def2 family of basis sets, is generally not appropriate for predicting NMR properties in heavy nuclei. These approaches replace the chemically inert core electrons with an effective potential and do not explicitly describe the wavefunction in the nuclear region, thereby compromising the description of the electronic density near the nucleus [82, 83]. As a result, chemical shifts significantly different from experimental values may be obtained [47, 84]. Although ECPs are generally reliable for geometric optimization, where relativistic effects have a reduced impact on the structure, their application to heavy-element NMR calculations should generally be avoided [47, 85].

In this context, the use of relativistic methods becomes indispensable for heavy nuclei, such as ^119^Sn, since relativistic effects directly influence the spectroscopic parameters. When combined with appropriate all-electron basis sets, relativistic Hamiltonians provide an explicit description of the electronic density in the nuclear region and are therefore suitable for heavy-element NMR calculations [47, 86, 87]. In the present work, scalar-relativistic effects were treated using the DKH2, ZORA, and spin-free X2C (SF-X2C-1e) Hamiltonians.

It is important to note that although the geometrical analysis identified levels of theory that more accurately reproduce the experimental crystallographic parameters, this performance cannot be assumed to extend to other molecular properties. Therefore, the NMR results were evaluated independently using all investigated levels of theory, without any preselection based on the geometric analysis, allowing an unbiased assessment of their relative performance.

The statistical parameters obtained from the comparison between experimental and calculated ^119^Sn NMR chemical shifts are presented in Table 3, while the complete statistical analysis, including 95% confidence intervals and *p*-values for the regression parameters, is provided in the Supporting Information (Table S11). The ^119^Sn chemical-shift range covered by the investigated systems represents a subset of the full ^119^Sn chemical-shift range, reflecting the specific chemical space considered in this study.

**Table 3:** Statistical parameters obtained from the comparison between experimental and calculated ^11^⁹Sn NMR chemical shifts with DKH2 [SARC-DKH-TZVP(Sn)/DKH-def2-TZVP(other atoms)], ZORA [SARC-ZORA-TZVP(Sn)/ZORA-def2-TZVP(other atoms)], and X2C [X2C-TZVPall-s] methods

	SARC-DKH-TZVP					SARC-ZORA-TZVP					X2C-TZVPALL-s				
Functional	*R*^2^	MD	MAD	RMSD	SD	*R*^2^	MD	MAD	RMSD	SD	*R*^2^	MD	MAD	RMSD	SD
B2PLYP-D3	0.975	48.64	82.83	92.68	82.74	0.975	49.33	78.63	88.10	76.56	0.975	45.73	87.12	99.73	92.95
B3LYP	0.968	179.16	179.16	192.05	72.57	0.968	177.79	177.79	192.30	76.88	0.985	− 91.26	91.26	101.29	46.10
BP86	0.967	180.18	180.18	190.86	66.03	0.967	178.73	178.73	190.73	69.83	0.986	− 90.09	90.09	97.37	38.73
CAM-B3LYP	0.970	177.04	177.04	190.57	73.96	0.970	175.79	175.79	191.05	78.48	0.986	− 88.11	88.11	99.74	49.00
M06-2X	0.972	180.86	180.86	195.24	77.13	0.972	179.23	179.23	195.48	81.82	0.984	− 86.89	90.07	100.08	52.09
M06-L	0.970	146.14	146.14	167.25	85.30	0.970	145.34	145.34	168.69	89.83	0.982	− 120.01	123.35	135.64	66.31
PBE	0.968	174.78	174.78	185.86	66.31	0.968	173.45	173.45	185.94	70.26	0.986	− 97.76	97.76	104.49	38.71
PBE0	0.972	166.47	166.47	179.88	71.48	0.972	165.42	165.42	180.59	75.99	0.987	− 101.23	101.23	110.25	45.81
r^2^SCAN	0.970	153.81	153.81	171.36	79.21	0.970	153.04	153.04	172.61	83.73	0.984	− 117.11	117.11	129.56	58.12
TPSS	0.971	160.52	160.52	174.00	70.42	0.971	159.68	159.68	174.88	74.79	0.987	− 109.61	109.61	117.52	44.46
ωB97M-V	0.973	167.33	167.33	182.86	77.36	0.973	166.72	166.72	184.13	81.97	0.985	− 94.72	95.59	107.63	53.60
ωB97X-D3	0.972	171.86	171.86	186.68	76.43	0.972	170.69	170.69	187.38	81.08	0.986	− 91.25	91.36	103.15	50.44

The average statistical parameters calculated across the twelve density functionals are summarized in Table 4.

**Table 4:** Average statistical parameters obtained for each relativistic Hamiltonian, computed over the 12 evaluated density functionals

Hamiltonian	*R*^2^_average_	MAD_average_ (ppm)	RMSD_average_ (ppm)	MD_average_ (ppm)	SD_average_ (ppm)
DKH	0.971	161.75	175.77	158.90	74.91
ZORA	0.971	160.38	175.99	157.93	78.43
X2C	0.984	98.55	108.87	− 86.86	53.03

Although all three Hamiltonians provide satisfactory correlations with the experimental data (average *R*^2^ ≥ 0.970), differences become evident when the prediction errors are analyzed. Among the evaluated relativistic approaches, X2C provides the lowest average MAD value (98.55 ppm), whereas DKH and ZORA exhibit very similar values of 161.75 and 160.38 ppm, respectively. Therefore, X2C yields an approximately 39% lower average absolute deviation than the scalar-relativistic approaches, indicating improved overall agreement with the experimental ^119^Sn chemical shifts for the investigated dataset.

A similar trend is observed for the correlation coefficients. While DKH and ZORA both provide an average *R*^2^ of 0.971, the X2C calculations increase this value to 0.984, indicating improved linear agreement between calculated and experimental chemical shifts. A similar improvement is observed for the RMSD values. The average RMSD decreases from 175.77 and 175.99 ppm for DKH and ZORA, respectively, to 108.87 ppm for X2C, indicating that X2C reduces both the average absolute deviation and the overall magnitude of the prediction errors within the investigated dataset.

A notable difference among the relativistic treatments concerns the systematic component of the error. Both DKH and ZORA exhibit positive average mean deviations (MD = 158.90 and 157.93 ppm, respectively), indicating a systematic overestimation of the experimental chemical shifts. For DKH and ZORA, approximately 97% of calculated shifts are higher than the experimental values, consistent with the positive MD. Conversely, X2C presents a negative average MD (− 86.86 ppm), indicating a change in the direction of the systematic deviation, with 92% of the calculated shifts being lower than the corresponding experimental values. Despite this inversion, DKH and ZORA show comparable average SD values (74.91 and 78.43 ppm, respectively), whereas X2C exhibits a lower average SD (53.03 ppm), indicating that the relativistic treatment affects not only the systematic contribution but also the overall distribution of deviations.

The dependence of the results on the exchange–correlation functional is similar for DKH and ZORA, but weaker for X2C. The difference between the lowest and highest MAD values is 98.03 ppm for DKH, 100.6 ppm for ZORA, and 36.23 ppm for X2C. Although these variations are smaller than the average absolute prediction errors, they demonstrate that the functional choice still contributes significantly to the final accuracy when combined with the relativistic treatment.

**Fig. 6:**
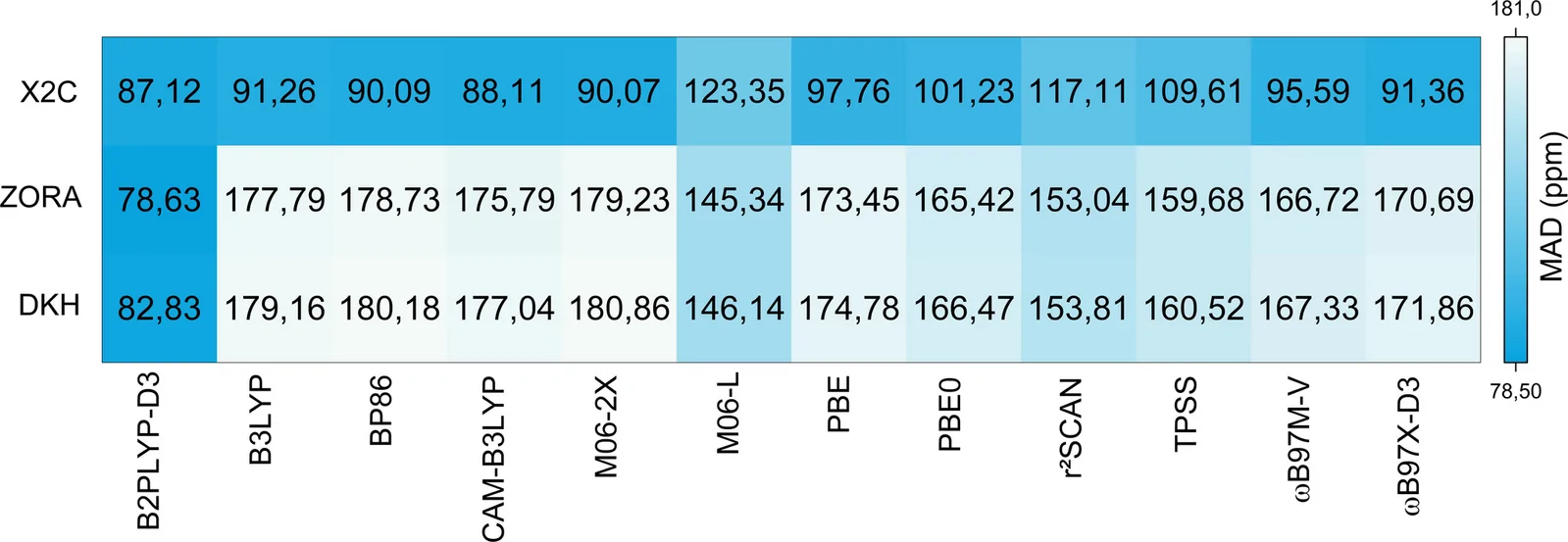
Heatmap summarizing the mean absolute deviations (MAD, ppm) for the calculated ^11^⁹Sn NMR chemical shifts obtained with the different computational protocols. The color scale represents the MAD values, with lower values corresponding to higher predictive accuracy

X2C provides the lowest absolute deviation for 6 compounds, and ZORA provides the lowest deviation for five compounds. DKH does not provide the lowest deviation for any compound. Although X2C does not outperform the scalar-relativistic approaches for every individual molecule, it provides the best overall agreement with the experimental data across the investigated dataset (Fig. 6).

These results show that the choice of relativistic Hamiltonian affects the calculated ^119^Sn NMR chemical shifts of R_2_Sn(dmit) complexes. While DKH and ZORA show comparable statistical performance, X2C provides lower average deviations and higher correlation coefficients within the present dataset. However, the statistical analysis alone does not establish the physical origin of the observed improvement, and the magnitude of the improvement depends on the density functional employed.

The statistical parameters obtained for the DKH method are presented in Table 3 (left panel). Among the 12 evaluated functionals, B2PLYP-D3 provides the best overall performance, with a MAD of 82.83 ppm and the lowest RMSD (92.68 ppm) among all functionals evaluated with DKH. This functional also presents the lowest MD (48.64 ppm) among the top performers, indicating that it combines good absolute accuracy with relatively low systematic bias. The M06-L functional ranks second with a MAD of 146.14 ppm and RMSD of 167.25 ppm, followed by r^2^SCAN (MAD = 153.81 ppm, RMSD = 171.36 ppm). Although B2PLYP-D3 does not have the lowest SD (82.74 ppm), its very low MD leads to the smallest RMSD, indicating the best overall agreement with experiment.

All functionals evaluated with DKH present positive MD values (ranging from 48.64 ppm for B2PLYP-D3 to 180.86 ppm for M06-2X), confirming that the method systematically overestimates the experimental chemical shifts regardless of the functional employed. This observation is consistent with the average MD of 158.90 ppm reported in Table 4. The regression slopes are close to or above unity for most functionals, except for B2PLYP-D3, which has a slope of 0.818 (95% CI 0.720–0.917), indicating compression of the calculated chemical shift range. For the other best-performing functionals, the slopes are moderately higher than unity (M06-L, 1.283; r^2^SCAN, 1.242), indicating some systematic expansion of the calculated chemical shift range, while intercepts are negative for all functionals, ranging from − 55.40 ppm (B2PLYP-D3) to − 203.1 ppm (M06-2X). Therefore, DKH introduces a systematic scaling error whose sign and magnitude depend on the functional employed.

The statistical parameters for the ZORA method are presented in Table 3 (center panel). Similar to DKH, B2PLYP-D3 provides the best overall performance, with a MAD of 78.63 ppm and the lowest RMSD (88.10 ppm) among all functionals evaluated with ZORA. Notably, B2PLYP-D3 exhibits a slightly lower MAD with ZORA compared to DKH (78.63 vs. 82.83 ppm), indicating a marginal improvement for this functional when ZORA is employed. The M06-L functional ranks second with a MAD of 145.34 ppm and RMSD of 168.69 ppm, followed by r^2^SCAN (MAD = 153.04 ppm, RMSD = 172.61 ppm). The ranking of the functionals remains essentially unchanged compared with DKH for the top positions, suggesting that both scalar-relativistic Hamiltonians provide a very similar description of the electronic structure.

Regression slopes for ZORA range from 0.836 (B2PLYP-D3) to 1.315 (M06-L), with slopes higher than 1.0 for most functionals, suggesting a slight amplification of the chemical shift range. The intercepts are negative for all functionals, ranging from − 55.3 ppm (B2PLYP-D3) to − 204.1 ppm (M06-2X). For ZORA/M06-L combination, the slope is 1.315, which deviates more significantly from 1.0 than the DKH/M06-L slope (1.283), indicating a slightly larger systematic scaling for ZORA.

The statistical parameters for the X2C method are presented in Table 3 (right panel). X2C provides the best overall performance among the three relativistic Hamiltonians, although its performance varies depending on the density functional employed.

The B2PLYP-D3 functional, combined with X2C, provides the lowest MAD (87.12 ppm) while BP86 gives the lowest RMSD (97.37 ppm). The M06-2X functional combined with X2C yields a MAD of 90.07 ppm and an RMSD of 100.08 ppm, which is substantially lower than DKH/M06-2X (MAD = 180.86 ppm, RMSD = 195.24 ppm) and ZORA/M06-2X (MAD = 179.23 ppm, RMSD = 195.48 ppm). CAM-B3LYP (MAD = 88.11 ppm, RMSD = 99.74 ppm) and ωB97X-D3 (MAD = 91.36 ppm, RMSD = 103.15 ppm) also yielded low MAD and RMSD values when combined with X2C.

B3LYP performs relatively poorly with both DKH and ZORA but improves substantially when combined with X2C. With a MAD of 91.26 ppm and RMSD of 101.29 ppm, B3LYP ranks 5th among the 12 functionals evaluated with X2C. This represents an improvement of approximately 87 ppm compared to ZORA (177.79 ppm) and DKH (179.16 ppm). The slope for X2C/B3LYP is 1.097, close to 1.0, suggesting a smaller systematic scaling bias, and the *R*^2^ of 0.985 indicates excellent correlation.

The M06-L functional, which was the second-best performer with DKH and ZORA, shows the highest MAD with X2C (MAD = 123.35 ppm, 12th place). Nevertheless, this still corresponds to a lower MAD than DKH/M06-L (146.14 ppm) and ZORA/M06-L (145.34 ppm), with an improvement of about 22 ppm. B2PLYP-D3 shows its highest MAD with X2C (MAD = 87.12 ppm, RMSD = 99.73 ppm), and is the only functional with a positive MD (45.73 ppm) in the X2C results. This indicates that B2PLYP-D3 is the only functional that still overestimates the chemical shifts when using X2C.

The PBE0 functional yields the highest *R*^2^ (0.987) among all evaluated combinations, with a MAD of 101.23 ppm and RMSD of 110.25 ppm. Although its MAD is slightly higher than that of the top-performing functionals, the correlation indicates that PBE0 captures the overall trend very well, even if individual deviations are slightly larger.

The improved performance observed for X2C suggests that relativistic treatment used here provides the best overall agreement with the experimental ^119^Sn NMR chemical shifts within the investigated dataset. However, the improvement obtained with X2C is not uniform for all complexes, demonstrating that the accuracy of ^119^Sn NMR predictions depends on the combined effect of the relativistic Hamiltonian, density functional, and molecular electronic structure.

The lowest MAD values among the evaluated protocols were obtained with ZORA/B2PLYP-D3 (78.63 ppm), followed by DKH/B2PLYP-D3 (82.83 ppm) and X2C/B2PLYP-D3 (87.12 ppm). Within X2C, the lowest MAD values were obtained with B2PLYP-D3 (87.12 ppm), CAM-B3LYP (88.11 ppm), M06-2X (90.07 ppm), and ωB97X-D3 (91.36 ppm). These combinations yielded MAD values below 100 ppm, *R*^2^ values between 0.975 and 0.986, and slopes ranging from 0.793 (B2PLYP-D3) to 1.132 (ωB97X-D3). These results indicate that, within the present dataset, with the exception of B2PLYP-D3, X2C combined with hybrid functionals provides the best overall numerical agreement with the experimental ^119^Sn chemical shifts. When B2PLYP-D3 is not computationally available or practical, several other combinations still provide good overall performance. Within X2C, the hybrid functionals CAM-B3LYP, M06-2X, and ωB97X-D3 provide competitive results (MAD = 88.11, 90.07, and 91.36 ppm, respectively). It is worth noting that the GGA functional BP86 performs comparably (MAD = 90.09 ppm), so the good performance is not exclusive to hybrid functionals. When considering scalar-relativistic approaches, ZORA/M06-L (MAD = 145.34 ppm) and DKH/M06-L (MAD = 146.14 ppm) represent the best alternatives, followed by ZORA/r^2^SCAN (MAD = 153.04 ppm) and DKH/r^2^SCAN (MAD = 153.81 ppm). These levels of theory may therefore represent suitable alternatives for studies requiring consistency with previous calculations while maintaining a reasonable computational cost.

Although relativistic effects are essential for describing heavy nuclei such as tin, the remaining deviations indicate that additional factors also influence the calculated chemical shifts. The presence of multiple Lewis resonance structures in the dmit ligand does not by itself establish significant multireference character of the electronic ground state. However, because wavefunction-based multireference diagnostics were not performed in the present study, possible static-correlation contributions cannot be quantitatively excluded. This represents an additional limitation of the present DFT-based analysis, particularly in the interpretation of the remaining deviations in the calculated ^11^⁹Sn NMR chemical shifts. Also, because SMD was originally parameterized using a limited set of exchange–correlation functionals, its performance may exhibit some functional dependence. Accordingly, differences among the NMR protocols considered here should be interpreted as reflecting the combined effects of the electronic-structure and solvation treatments rather than being attributed exclusively to the density functional.

A comprehensive benchmark study on ^119^Sn NMR chemical shifts reported that state-of-the-art computational protocols can achieve mean absolute deviations below 100 ppm for a chemically diverse set of organotin compounds [47]. However, the SnS51 benchmark does not include organotin complexes containing the dmit ligand, and therefore direct comparison between the two datasets should be made with caution. The R₂Sn(dmit) complexes investigated here present a distinct coordination environment around the tin center, characterized by sulfur-rich ligands and a highly polarizable electronic structure, which may increase the difficulty of accurately reproducing the experimental ^119^Sn chemical shifts. Consequently, the larger MAD values obtained in the present work (ranging from approximately 79 to 181 ppm depending on the level of theory, with the best X2C protocols reaching below 100 ppm) should be interpreted in the context of the specific chemical space investigated rather than as evidence of a general limitation of the computational methods. Therefore, considering the limited size and diversity of the dataset, the conclusions presented here should be restricted to the R₂Sn(dmit) complexes investigated in this study. Nevertheless, the results demonstrate the importance of systematically evaluating both relativistic Hamiltonians and exchange–correlation functionals when selecting computational protocols for the prediction of ^119^Sn NMR chemical shifts in the investigated organotin compounds.

## Conclusions

A systematic evaluation of density functional theory approaches was conducted with the aim of identifying reliable computational protocols for describing the structural and spectroscopic properties of R₂Sn(dmit) complexes.

For geometric optimization, the choice of exchange–correlation functional proved to be more influential than the size of the basis set. The PBE/def2-SVP and BP86/def2-SVP combinations yielded the smallest global statistical deviations, while M06-2X/def2-TZVP was the best-performing hybrid functional. The B3LYP/def2-SVP combination also performed competitively, offering an alternative when the priority is to minimize systematic deviations. Increasing the basis set size from def2-SVP to def2-TZVP and def2-QZVP yielded only minimal improvements, indicating that the def2-SVP basis set can provide a favorable balance between accuracy and computational cost for geometry optimization within the investigated dataset.

Vibrational analysis confirmed this trend. After applying linear scaling, the BP86 and PBE functionals showed the best agreement with the experimental infrared frequencies, while larger basis sets did not yield systematic improvements. These results reinforce the conclusion that increasing the basis set beyond the triple-ζ level does not provide systematic improvements in the calculated vibrational frequencies.

The choice of relativistic treatment had a pronounced influence on the calculated ^119^Sn chemical shifts. Among the evaluated levels of theory, B2PLYP-D3/ZORA and B2PLYP-D3/DKH yielded the best overall agreement with the experimental data (MAD = 78.63 and 82.83 ppm, respectively) within the investigated dataset. When B2PLYP-D3 is computationally impractical, X2C combined with CAM-B3LYP (88.11 ppm), M06-2X (90.07 ppm), or ωB97X-D3 (91.36 ppm) provides a competitive alternative; BP86/X2C also shows comparable performance at a lower functional complexity. Also M06-L/ZORA (145.34 ppm) and M06-L/DKH (146.14 ppm) provide comparable results, and may serve as practical alternatives when X2C calculations are not available. These results highlight the importance of an appropriate relativistic treatment for improving the prediction of ^119^Sn NMR chemical shifts.

Overall, this comparative analysis demonstrates that the most appropriate computational protocol within the investigated dataset depends on the molecular property under investigation. For geometry optimization and vibrational frequencies, computationally efficient GGA functionals combined with relatively modest basis sets provided a favorable balance between accuracy and computational cost within the investigated dataset. In contrast, ^11^⁹Sn NMR chemical shifts showed a stronger dependence on the relativistic treatment, with X2C providing the best overall agreement with the available experimental data for several hybrid functionals. Nevertheless, given the remaining absolute errors, the limited size and chemical diversity of the dataset, and the absence of an independent external-validation set, these conclusions should be interpreted within the specific chemical space investigated in this study.

## Supplementary Information

Below is the link to the electronic supplementary material.

ESM 1 (DOCX 236 KB)

## Data Availability

The calculated data and the experimental reference values used in the analyses are provided in the article and its Supplementary Information.
