# Supplementary material for: Systematic investigation of density functional theory methods for diorganotin 1,3-dithiole-2-thione-4,5-dithiolate complexes, R2Sn(dmit)
Source: J Mol Model. 2026 Oct 13;32(11):416. doi: 10.1007/s00894-026-06990-3 (PMC13661709; doi:10.1007/s00894-026-06990-3)
Supplement: Supplementary file 1 [file 894_2026_6990_MOESM1_ESM.docx]

**Supplementary Information**

**Systematic Investigation of Density Functional Theory Methods for Diorganotin 1,3-dithiole-2-thione-4,5-dithiolate Complexes, R_2_Sn(dmit)**

Raíssa Ribeiro Domingues Brum^1^, Jonathan B. Brum^1†^_,_ Glaucio Braga Ferreira^1,2^*^†^

^1*^Graduate Program in Chemistry, Chemistry Institute, Fluminense Federal University, Outeiro de S. João Batista s/n., Centro, Niterói, 24210-130, Rio de Janeiro, Brazil.

^2^Department of Inorganic Chemistry, Chemistry Institute, Fluminense Federal University, Outeiro de S. João Batista s/n., Centro, Niterói, 24210-130, Rio de Janeiro, Brazil.

*Corresponding author(s). E-mail(s): [glauciobf@id.uff.br](mailto:glauciobf@id.uff.br);

Contributing authors: [raribeiro@id.uff.br](mailto:raribeiro@id.uff.br); [jonathanbrito@id.uff.br](mailto:jonathanbrito@id.uff.br);

†These authors contributed equally to this work.

**Figures**

Figure S1 Molecular structures of the four additional tin compounds included in the NMR analysis. a) C_26_H_24_SSn [1] ; b) C_6_H_12_N_2_S_3_Sn [2] ; c) C_16_H_25_N_3_S_3_Sn [2]; d) C_21_H_35_N_3_S_6_Sn[3].

**Tables**

Table S1 – Statistical evaluation of the calculated ^119^Sn NMR chemical shifts obtained with DKH2 [SARC-DKH-TZVP(Sn)/DKH-def2-TZVP(other atoms)], ZORA [SARC-ZORA-TZVP(Sn)/ZORA-def2-TZVP(other atoms)], and X2C [X2C-TZVPall-s] comparing the results obtained using the lowest-energy conformer and Boltzmann-weighted chemical shifts

| Functional | R² | R² lowconf | MAD | MAD lowconf | Absolute difference | Relative difference (%) |
| --- | --- | --- | --- | --- | --- | --- |
| SARC-DKH-TZVP | | | | | | |
| B2PLYP-D3 | 0.952 | 0.952 | 85.30 | 86.60 | 1.30 | 1.50 |
| B3LYP | 0.904 | 0.903 | 177.40 | 177.50 | 0.10 | 0.06 |
| BP86 | 0.900 | 0.900 | 178.20 | 178.60 | 0.40 | 0.22 |
| CAM-B3LYP | 0.910 | 0.910 | 177.20 | 177.30 | 0.10 | 0.06 |
| M06-2X | 0.919 | 0.918 | 178.40 | 178.60 | 0.20 | 0.11 |
| M06-L | 0.910 | 0.910 | 144.80 | 145.00 | 0.20 | 0.14 |
| PBE | 0.904 | 0.903 | 173.00 | 173.40 | 0.40 | 0.23 |
| PBE0 | 0.916 | 0.915 | 165.80 | 166.10 | 0.30 | 0.18 |
| r²SCAN | 0.909 | 0.908 | 153.00 | 153.30 | 0.30 | 0.20 |
| TPSS | 0.913 | 0.913 | 159.40 | 159.80 | 0.40 | 0.25 |
| ωB97M-V | 0.920 | 0.920 | 168.30 | 168.20 | 0.10 | 0.06 |
| ωB97X-D3 | 0.916 | 0.916 | 172.30 | 172.50 | 0.20 | 0.12 |
|  | | | | | | |
|  | | | | | | |
| SARC-ZORA-TZVP | | | | | | |
| B2PLYP-D3 | 0.952 | 0.952 | 82.70 | 84.00 | 1.30 | 1.55 |
| B3LYP | 0.903 | 0.902 | 175.40 | 175.60 | 0.20 | 0.11 |
| BP86 | 0.900 | 0.899 | 176.10 | 176.50 | 0.40 | 0.23 |
| CAM-B3LYP | 0.909 | 0.909 | 175.20 | 175.40 | 0.20 | 0.11 |
| M06-2X | 0.918 | 0.918 | 176.30 | 176.50 | 0.20 | 0.11 |
| M06-L | 0.910 | 0.909 | 143.30 | 143.50 | 0.20 | 0.14 |
| PBE | 0.903 | 0.902 | 171.00 | 171.50 | 0.50 | 0.29 |
| PBE0 | 0.915 | 0.914 | 164.10 | 164.40 | 0.30 | 0.18 |
| r²SCAN | 0.908 | 0.908 | 151.40 | 151.80 | 0.40 | 0.26 |
| TPSS | 0.913 | 0.912 | 157.90 | 158.20 | 0.30 | 0.19 |
| ωB97M-V | 0.920 | 0.919 | 166.60 | 166.50 | 0.10 | 0.06 |
| ωB97X-D3 | 0.916 | 0.915 | 170.50 | 170.60 | 0.10 | 0.06 |
| X2C-TZVPall-s | | | | | | |
| B2PLYP-D3 | 0.945 | 0.946 | 88.10 | 89.20 | 1.10 | 1.23 |
| B3LYP | 0.970 | 0.973 | 88.10 | 86.50 | 1.60 | 1.85 |
| BP86 | 0.968 | 0.971 | 86.20 | 84.50 | 1.70 | 2.01 |
| CAM-B3LYP | 0.972 | 0.975 | 83.40 | 81.80 | 1.60 | 1.96 |
| M06-2X | 0.980 | 0.982 | 82.20 | 80.50 | 1.70 | 2.11 |
| M06-L | 0.960 | 0.965 | 115.70 | 114.60 | 1.10 | 0.96 |
| PBE | 0.970 | 0.973 | 93.60 | 91.90 | 1.70 | 1.85 |
| PBE0 | 0.975 | 0.978 | 96.70 | 94.90 | 1.80 | 1.90 |
| r²SCAN | 0.958 | 0.963 | 113.70 | 112.20 | 1.50 | 1.34 |
| TPSS | 0.973 | 0.977 | 105.10 | 103.40 | 1.70 | 1.64 |
| ωB97M-V | 0.974 | 0.978 | 88.10 | 86.50 | 1.60 | 1.85 |
| ωB97X-D3 | 0.974 | 0.978 | 85.90 | 84.40 | 1.50 | 1.78 |

*lowconf = lower-energy conformer

Table S2 - Experimental structural parameters of the R_2_Sn(dmit) complexes used as reference for the evaluation of the calculated geometries. [4–11]

| Structure | Sn - S_1_ (Å) | Sn - S_2_ (Å) | C - Sn – C (°) |
| --- | --- | --- | --- |
| Me_2_Sn(dmit) | 2.440 | 2.518 | 124.9 |
| Me_2_Sn(dmit) | 2.457 | 2.521 | 126.0 |
| MePhSn(dmit) | 2.437 | 2.487 | 115.8 |
| Ph_2_Sn(dmit) | 2.455 | 2.533 | 120.5 |
| Et_2_Sn(dmit) | 2.450 | 2.460 | 126.3 |
| Et_2_Sn(dmit) | 2.448 | 2.457 | 119.5 |
| Et_2_Sn(dmit) | 2.459 | 2.524 | 130.8 |
| Et_2_Sn(dmit) | 2.436 | 2.513 | 128.7 |
| t-Bu_2_Sn(dmit) | 2.463 | 2.534 | 120.9 |
| t-Bu_2_Sn(dmit) | 2.445 | 2.533 | 121.4 |
| (C_4_H_7_O_2_)_2_Sn(dmit) | 2.471 | 2.471 | 121.1 |
| (C_6_H_11_O)_2_Sn(dmit) | 2.492 | 2.502 | 131.2 |
| (C_5_H_9_O_2_)_2_Sn(dmit) | 2.481 | 2.496 | 130.0 |
| (C_3_H_6_NO)_2_Sn(dmit) | 2.507 | 2.524 | 129.1 |

Table S3 - Calculated bond lengths and bond angles for the R_2_Sn(dmit) complexes obtained using different levels of theory.

|  |  | def2-SVP | | | def2-TZVP | | | def2-QZVP | | |
| --- | --- | --- | --- | --- | --- | --- | --- | --- | --- | --- |
| DFT | Structures | Sn – S_1_ (Å) | Sn – S_2_ (Å) | C - Sn - C (°) | Sn – S_1_ (Å) | Sn – S_2_ (Å) | C - Sn - C (°) | Sn – S_1_ (Å) | Sn – S_2_ (Å) | C - Sn - C (°) |
| PBE | Me_2_Sn(dmit) | 2.476 | 2.476 | 115.6 | 2.451 | 2.451 | 114.7 | 2.451 | 2.451 | 114.8 |
|  | MePhSn(dmit) | 2.474 | 2.480 | 114.2 | 2.452 | 2.453 | 113.6 | 2.453 | 2.454 | 113.5 |
|  | Ph_2_Sn(dmit) | 2.479 | 2.479 | 113.8 | 2.454 | 2.454 | 113.0 | 2.454 | 2.454 | 112.9 |
|  | Et_2_Sn(dmit) | 2.482 | 2.485 | 118.7 | 2.458 | 2.460 | 117.0 | 2.458 | 2.460 | 117.1 |
|  | t-Bu_2_Sn(dmit) | 2.495 | 2.495 | 124.2 | 2.472 | 2.472 | 122.7 | 2.472 | 2.472 | 122.7 |
|  | (C_4_H_7_O_2_)_2_Sn(dmit) | 2.503 | 2.507 | 133.1 | 2.480 | 2.485 | 130.7 | 2.481 | 2.487 | 131.1 |
|  | (C_6_H_11_O)_2_Sn(dmit) | 2.508 | 2.555 | 137.5 | 2.483 | 2.534 | 134.5 | 2.483 | 2.535 | 134.5 |
|  | (C_5_H_9_O_2_)_2_Sn(dmit) | 2.485 | 2.485 | 120.5 | 2.461 | 2.461 | 118.1 | 2.461 | 2.461 | 118.0 |
|  | (C_3_H_6_NO)_2_Sn(dmit) | 2.528 | 2.547 | 107.7 | 2.498 | 2.531 | 111.0 | 2.499 | 2.535 | 111.4 |
| BP86 | Me_2_Sn(dmit) | 2.480 | 2.480 | 115.5 | 2.455 | 2.455 | 114.6 | 2.455 | 2.455 | 114.7 |
|  | MePhSn(dmit) | 2.479 | 2.484 | 114.4 | 2.457 | 2.458 | 113.8 | 2.457 | 2.458 | 113.7 |
|  | Ph_2_Sn(dmit) | 2.483 | 2.483 | 113.9 | 2.459 | 2.459 | 113.1 | 2.458 | 2.458 | 113.1 |
|  | Et_2_Sn(dmit) | 2.487 | 2.489 | 119.0 | 2.463 | 2.464 | 117.5 | 2.463 | 2.464 | 117.5 |
|  | t-Bu_2_Sn(dmit) | 2.501 | 2.501 | 124.9 | 2.478 | 2.478 | 123.5 | 2.478 | 2.478 | 123.5 |
|  | (C_4_H_7_O_2_)_2_Sn(dmit) | 2.508 | 2.513 | 133.3 | 2.485 | 2.491 | 131.3 | 2.486 | 2.492 | 131.6 |
|  | (C_6_H_11_O)_2_Sn(dmit) | 2.512 | 2.561 | 137.2 | 2.488 | 2.540 | 134.6 | 2.487 | 2.541 | 134.6 |
|  | (C_5_H_9_O_2_)_2_Sn(dmit) | 2.489 | 2.489 | 119.0 | 2.465 | 2.465 | 117.0 | 2.464 | 2.464 | 117.0 |
|  | (C_3_H_6_NO)_2_Sn(dmit) | 2.533 | 2.552 | 107.5 | 2.499 | 2.533 | 109.7 | 2.499 | 2.534 | 109.4 |

Table S3 - Continued

|  |  | def2-SVP | | | def2-TZVP | | | def2-QZVP | | |
| --- | --- | --- | --- | --- | --- | --- | --- | --- | --- | --- |
| DFT | Structures | Sn – S_1_ (Å) | Sn – S_2_ (Å) | C - Sn - C (°) | Sn – S_1_ (Å) | Sn – S_2_ (Å) | C - Sn - C (°) | Sn – S_1_ (Å) | Sn – S_2_ (Å) | C - Sn - C (°) |
| M06-L | Me_2_Sn(dmit) | 2.467 | 2.468 | 115.9 | 2.436 | 2.438 | 114.3 | 2.435 | 2.436 | 114.7 |
|  | MePhSn(dmit) | 2.468 | 2.468 | 113.8 | 2.439 | 2.440 | 112.3 | 2.438 | 2.438 | 112.6 |
|  | Ph_2_Sn(dmit) | 2.468 | 2.470 | 114.3 | 2.440 | 2.441 | 112.3 | 2.438 | 2.439 | 112.3 |
|  | Et_2_Sn(dmit) | 2.470 | 2.475 | 116.9 | 2.440 | 2.446 | 114.7 | 2.438 | 2.443 | 113.2 |
|  | t-Bu_2_Sn(dmit) | 2.481 | 2.482 | 124.1 | 2.453 | 2.454 | 122.4 | 2.451 | 2.452 | 122.5 |
|  | (C_4_H_7_O_2_)_2_Sn(dmit) | 2.467 | 2.513 | 127.1 | 2.444 | 2.490 | 127.9 | 2.444 | 2.493 | 127.6 |
|  | (C_6_H_11_O)_2_Sn(dmit) | 2.493 | 2.543 | 135.6 | 2.464 | 2.511 | 134.2 | 2.461 | 2.512 | 134.5 |
|  | (C_5_H_9_O_2_)_2_Sn(dmit) | 2.483 | 2.484 | 125.3 | 2.454 | 2.457 | 123.8 | 2.455 | 2.456 | 124.9 |
|  | (C_3_H_6_NO)_2_Sn(dmit) | 2.535 | 2.541 | 111.1 | 2.505 | 2.520 | 111.3 | 2.502 | 2.520 | 111.5 |
| TPSS | Me_2_Sn(dmit) | 2.473 | 2.473 | 115.8 | 2.446 | 2.446 | 114.6 | 2.446 | 2.446 | 114.6 |
|  | MePhSn(dmit) | 2.471 | 2.476 | 114.5 | 2.447 | 2.449 | 113.6 | 2.448 | 2.449 | 113.5 |
|  | Ph_2_Sn(dmit) | 2.476 | 2.476 | 114.3 | 2.450 | 2.450 | 113.1 | 2.449 | 2.449 | 113.0 |
|  | Et_2_Sn(dmit) | 2.479 | 2.482 | 118.6 | 2.453 | 2.454 | 117.1 | 2.453 | 2.454 | 117.1 |
|  | t-Bu_2_Sn(dmit) | 2.492 | 2.492 | 124.8 | 2.467 | 2.467 | 123.5 | 2.466 | 2.467 | 123.1 |
|  | (C_4_H_7_O_2_)_2_Sn(dmit) | 2.504 | 2.508 | 133.7 | 2.480 | 2.486 | 132.0 | 2.480 | 2.487 | 132.1 |
|  | (C_6_H_11_O)_2_Sn(dmit) | 2.508 | 2.558 | 139.0 | 2.481 | 2.534 | 136.0 | 2.480 | 2.535 | 135.9 |
|  | (C_5_H_9_O_2_)_2_Sn(dmit) | 2.482 | 2.482 | 119.7 | 2.456 | 2.456 | 117.6 | 2.455 | 2.455 | 117.3 |
|  | (C_3_H_6_NO)_2_Sn(dmit) | 2.538 | 2.542 | 108.3 | 2.509 | 2.530 | 109.3 | 2.509 | 2.532 | 109.3 |

Table S3 - Continued

|  |  | def2-SVP | | | def2-TZVP | | | def2-QZVP | | |
| --- | --- | --- | --- | --- | --- | --- | --- | --- | --- | --- |
| DFT | Structures | Sn – S_1_ (Å) | Sn – S_2_ (Å) | C - Sn - C (°) | Sn – S_1_ (Å) | Sn – S_2_ (Å) | C - Sn - C (°) | Sn – S_1_ (Å) | Sn – S_2_ (Å) | C - Sn - C (°) |
| r^2^SCAN | Me_2_Sn(dmit) | 2.458 | 2.458 | 116.1 | 2.431 | 2.431 | 114.8 | 2.431 | 2.431 | 114.9 |
|  | MePhSn(dmit) | 2.458 | 2.459 | 114.6 | 2.433 | 2.433 | 113.4 | 2.432 | 2.432 | 113.4 |
|  | Ph_2_Sn(dmit) | 2.459 | 2.459 | 113.3 | 2.434 | 2.434 | 112.1 | 2.432 | 2.433 | 112.2 |
|  | Et_2_Sn(dmit) | 2.463 | 2.466 | 118.9 | 2.437 | 2.439 | 115.9 | 2.436 | 2.439 | 115.9 |
|  | t-Bu_2_Sn(dmit) | 2.473 | 2.474 | 124.3 | 2.448 | 2.449 | 122.0 | 2.447 | 2.448 | 122.0 |
|  | (C_4_H_7_O_2_)_2_Sn(dmit) | 2.463 | 2.512 | 130.1 | 2.437 | 2.489 | 126.1 | 2.436 | 2.489 | 126.1 |
|  | (C_6_H_11_O)_2_Sn(dmit) | 2.486 | 2.538 | 137.8 | 2.460 | 2.517 | 134.9 | 2.459 | 2.518 | 134.9 |
|  | (C_5_H_9_O_2_)_2_Sn(dmit) | 2.473 | 2.474 | 126.2 | 2.442 | 2.442 | 120.9 | 2.443 | 2.443 | 122.4 |
|  | (C_3_H_6_NO)_2_Sn(dmit) | 2.525 | 2.528 | 109.4 | 2.497 | 2.515 | 109.7 | 2.497 | 2.516 | 109.8 |
| B3LYP | Me_2_Sn(dmit) | 2.473 | 2.473 | 115.9 | 2.450 | 2.450 | 115.5 | 2.451 | 2.451 | 114.7 |
|  | MePhSn(dmit) | 2.473 | 2.477 | 114.0 | 2.453 | 2.454 | 113.9 | 2.452 | 2.453 | 113.5 |
|  | Ph_2_Sn(dmit) | 2.475 | 2.476 | 113.3 | 2.453 | 2.453 | 113.7 | 2.453 | 2.453 | 112.9 |
|  | Et_2_Sn(dmit) | 2.480 | 2.483 | 118.3 | 2.458 | 2.459 | 116.5 | 2.458 | 2.460 | 117.2 |
|  | t-Bu_2_Sn(dmit) | 2.492 | 2.493 | 122.9 | 2.471 | 2.473 | 121.7 | 2.472 | 2.472 | 121.9 |
|  | (C_4_H_7_O_2_)_2_Sn(dmit) | 2.485 | 2.527 | 132.4 | 2.457 | 2.506 | 126.5 | 2.461 | 2.511 | 128.4 |
|  | (C_6_H_11_O)_2_Sn(dmit) | 2.500 | 2.550 | 134.9 | 2.477 | 2.530 | 132.5 | 2.479 | 2.534 | 132.6 |
|  | (C_5_H_9_O_2_)_2_Sn(dmit) | 2.483 | 2.484 | 119.0 | 2.458 | 2.461 | 115.1 | 2.460 | 2.460 | 115.8 |
|  | (C_3_H_6_NO)_2_Sn(dmit) | 2.516 | 2.542 | 107.9 | 2.461 | 2.532 | 119.2 | 2.459 | 2.529 | 119.3 |

Table S3 - Continued

|  |  | def2-SVP | | | def2-TZVP | | | def2-QZVP | | |
| --- | --- | --- | --- | --- | --- | --- | --- | --- | --- | --- |
| DFT | Structures | Sn – S_1_ (Å) | Sn – S_2_ (Å) | C - Sn - C (°) | Sn – S_1_ (Å) | Sn – S_2_ (Å) | C - Sn - C (°) | Sn – S_1_ (Å) | Sn – S_2_ (Å) | C - Sn - C (°) |
| PBE0 | Me_2_Sn(dmit) | 2.449 | 2.449 | 115.8 | 2.424 | 2.424 | 114.7 | 2.423 | 2.423 | 114.8 |
|  | MePhSn(dmit) | 2.447 | 2.451 | 114.3 | 2.424 | 2.425 | 113.5 | 2.424 | 2.425 | 113.5 |
|  | Ph_2_Sn(dmit) | 2.449 | 2.449 | 113.8 | 2.425 | 2.425 | 112.8 | 2.424 | 2.424 | 112.7 |
|  | Et_2_Sn(dmit) | 2.453 | 2.456 | 118.5 | 2.428 | 2.431 | 116.6 | 2.429 | 2.430 | 116.9 |
|  | t-Bu_2_Sn(dmit) | 2.463 | 2.464 | 123.7 | 2.440 | 2.440 | 121.9 | 2.440 | 2.440 | 122.0 |
|  | (C_4_H_7_O_2_)_2_Sn(dmit) | 2.460 | 2.503 | 133.3 | 2.432 | 2.482 | 127.8 | 2.431 | 2.483 | 127.7 |
|  | (C_6_H_11_O)_2_Sn(dmit) | 2.474 | 2.525 | 136.5 | 2.450 | 2.504 | 133.6 | 2.449 | 2.505 | 133.6 |
|  | (C_5_H_9_O_2_)_2_Sn(dmit) | 2.457 | 2.457 | 120.3 | 2.432 | 2.432 | 117.9 | 2.432 | 2.432 | 117.8 |
|  | (C_3_H_6_NO)_2_Sn(dmit) | 2.504 | 2.515 | 109.0 | 2.465 | 2.507 | 111.3 | 2.467 | 2.508 | 111.2 |
| CAM-B3LYP | Me_2_Sn(dmit) | 2.444 | 2.444 | 116.4 | 2.422 | 2.422 | 115.8 | 2.423 | 2.423 | 114.7 |
|  | MePhSn(dmit) | 2.443 | 2.447 | 114.0 | 2.422 | 2.425 | 113.4 | 2.423 | 2.424 | 113.2 |
|  | Ph_2_Sn(dmit) | 2.444 | 2.446 | 113.1 | 2.423 | 2.423 | 113.4 | 2.423 | 2.423 | 112.6 |
|  | Et_2_Sn(dmit) | 2.448 | 2.452 | 118.2 | 2.427 | 2.430 | 115.6 | 2.427 | 2.430 | 116.8 |
|  | t-Bu_2_Sn(dmit) | 2.458 | 2.458 | 122.4 | 2.438 | 2.439 | 121.0 | 2.438 | 2.438 | 120.6 |
|  | (C_4_H_7_O_2_)_2_Sn(dmit) | 2.455 | 2.499 | 132.2 | 2.427 | 2.479 | 125.2 | 2.428 | 2.482 | 126.5 |
|  | (C_6_H_11_O)_2_Sn(dmit) | 2.466 | 2.518 | 134.7 | 2.443 | 2.499 | 131.7 | 2.446 | 2.503 | 132.3 |
|  | (C_5_H_9_O_2_)_2_Sn(dmit) | 2.452 | 2.453 | 116.8 | 2.432 | 2.432 | 116.5 | 2.431 | 2.431 | 116.7 |
|  | (C_3_H_6_NO)_2_Sn(dmit) | 2.490 | 2.511 | 107.3 | 2.428 | 2.500 | 120.2 | 2.429 | 2.501 | 119.9 |

Table S3 - Continued

|  |  | def2-SVP | | | def2-TZVP | | | def2-QZVP | | |
| --- | --- | --- | --- | --- | --- | --- | --- | --- | --- | --- |
| DFT | Structures | Sn – S_1_ (Å) | Sn – S_2_ (Å) | C - Sn - C (°) | Sn – S_1_ (Å) | Sn – S_2_ (Å) | C - Sn - C (°) | Sn – S_1_ (Å) | Sn – S_2_ (Å) | C - Sn - C (°) |
| M06-2X | Me_2_Sn(dmit) | 2.457 | 2.459 | 118.6 | 2.435 | 2.436 | 117.9 | 2.436 | 2.437 | 115.2 |
|  | MePhSn(dmit) | 2.457 | 2.460 | 113.9 | 2.436 | 2.437 | 113.4 | 2.436 | 2.436 | 112.8 |
|  | Ph_2_Sn(dmit) | 2.457 | 2.460 | 112.7 | 2.437 | 2.437 | 112.8 | 2.436 | 2.436 | 112.1 |
|  | Et_2_Sn(dmit) | 2.460 | 2.466 | 118.2 | 2.440 | 2.446 | 115.1 | 2.439 | 2.443 | 114.5 |
|  | t-Bu_2_Sn(dmit) | 2.469 | 2.473 | 124.2 | 2.449 | 2.457 | 119.9 | 2.451 | 2.451 | 119.9 |
|  | (C_4_H_7_O_2_)_2_Sn(dmit) | 2.466 | 2.511 | 130.1 | 2.438 | 2.495 | 125.0 | 2.491 | 2.491 | 125.3 |
|  | (C_6_H_11_O)_2_Sn(dmit) | 2.480 | 2.534 | 136.6 | 2.457 | 2.511 | 132.6 | 2.513 | 2.513 | 133.4 |
|  | (C_5_H_9_O_2_)_2_Sn(dmit) | 2.474 | 2.477 | 126.2 | 2.454 | 2.455 | 120.9 | 2.455 | 2.455 | 123.8 |
|  | (C_3_H_6_NO)_2_Sn(dmit) | 2.513 | 2.526 | 108.4 | 2.495 | 2.515 | 107.1 | 2.501 | 2.517 | 109.9 |
| ωB97X-D3 | Me_2_Sn(dmit) | 2.438 | 2.439 | 116.1 | 2.412 | 2.413 | 116.3 | 2.414 | 2.414 | 115.7 |
|  | MePhSn(dmit) | 2.437 | 2.439 | 114.5 | 2.413 | 2.413 | 113.6 | 2.414 | 2.414 | 114.0 |
|  | Ph_2_Sn(dmit) | 2.435 | 2.438 | 114.9 | 2.412 | 2.412 | 112.4 | 2.412 | 2.412 | 112.2 |
|  | Et_2_Sn(dmit) | 2.440 | 2.445 | 117.9 | 2.416 | 2.420 | 116.0 | 2.416 | 2.420 | 115.8 |
|  | t-Bu_2_Sn(dmit) | 2.445 | 2.451 | 123.5 | 2.427 | 2.428 | 122.4 | 2.425 | 2.427 | 121.3 |
|  | (C_4_H_7_O_2_)_2_Sn(dmit) | 2.439 | 2.487 | 127.0 | 2.414 | 2.464 | 123.8 | 2.415 | 2.466 | 125.7 |
|  | (C_6_H_11_O)_2_Sn(dmit) | 2.455 | 2.505 | 133.8 | 2.432 | 2.486 | 131.8 | 2.434 | 2.489 | 134.1 |
|  | (C_5_H_9_O_2_)_2_Sn(dmit) | 2.453 | 2.454 | 125.0 | 2.428 | 2.431 | 123.1 | 2.430 | 2.430 | 123.6 |
|  | (C_3_H_6_NO)_2_Sn(dmit) | 2.490 | 2.519 | 111.5 | 2.418 | 2.494 | 120.9 | 2.461 | 2.506 | 113.2 |

Table S3 - Continued

|  |  | def2-SVP | | | def2-TZVP | | | def2-QZVP | | |
| --- | --- | --- | --- | --- | --- | --- | --- | --- | --- | --- |
| DFT | Structures | Sn – S_1_ (Å) | Sn – S_2_ (Å) | C - Sn - C (°) | Sn – S_1_ (Å) | Sn – S_2_ (Å) | C - Sn - C (°) | Sn – S_1_ (Å) | Sn – S_2_ (Å) | C - Sn - C (°) |
| ωB97M-V | Me_2_Sn(dmit) | 2.434 | 2.434 | 117.2 | 2.413 | 2.413 | 116.4 | 2.414 | 2.414 | 115.7 |
|  | MePhSn(dmit) | 2.433 | 2.436 | 114.5 | 2.432 | 2.436 | 114.8 | 2.414 | 2.414 | 114.0 |
|  | Ph_2_Sn(dmit) | 2.433 | 2.433 | 114.3 | 2.412 | 2.414 | 113.5 | 2.412 | 2.412 | 112.2 |
|  | Et_2_Sn(dmit) | 2.436 | 2.442 | 118.6 | 2.416 | 2.420 | 115.5 | 2.416 | 2.420 | 115.8 |
|  | t-Bu_2_Sn(dmit) | 2.444 | 2.446 | 123.9 | 2.425 | 2.428 | 121.0 | 2.425 | 2.427 | 121.3 |
|  | (C_4_H_7_O_2_)_2_Sn(dmit) | 2.439 | 2.485 | 131.1 | 2.416 | 2.467 | 125.9 | 2.415 | 2.466 | 125.7 |
|  | (C_6_H_11_O)_2_Sn(dmit) | 2.453 | 2.501 | 135.9 | 2.432 | 2.485 | 133.5 | 2.434 | 2.489 | 134.1 |
|  | (C_5_H_9_O_2_)_2_Sn(dmit) | 2.450 | 2.450 | 125.5 | 2.430 | 2.431 | 124.2 | 2.430 | 2.431 | 123.6 |
|  | (C_3_H_6_NO)_2_Sn(dmit) | 2.492 | 2.513 | 111.4 | 2.462 | 2.509 | 113.7 | 2.462 | 2.506 | 113.2 |
| B2PLYP-D3 | Me_2_Sn(dmit) | 2.454 | 2.455 | 116.1 | 2.454 | 2.455 | 116.1 | 2.428 | 2.428 | 115.6 |
|  | MePhSn(dmit) | 2.453 | 2.456 | 114.5 | 2.453 | 2.456 | 114.5 | 2.428 | 2.428 | 113.8 |
|  | Ph_2_Sn(dmit) | 2.453 | 2.455 | 112.8 | 2.453 | 2.455 | 112.8 | 2.427 | 2.427 | 112.2 |
|  | Et_2_Sn(dmit) | 2.457 | 2.461 | 117.7 | 2.432 | 2.435 | 117.3 | 2.432 | 2.434 | 117.2 |
|  | t-Bu_2_Sn(dmit) | 2.465 | 2.466 | 123.9 | 2.465 | 2.466 | 123.9 | 2.441 | 2.442 | 122.9 |
|  | (C_4_H_7_O_2_)_2_Sn(dmit) | 2.455 | 2.500 | 127.4 | 2.431 | 2.479 | 124.0 | 2.430 | 2.478 | 125.7 |
|  | (C_6_H_11_O)_2_Sn(dmit) | 2.474 | 2.523 | 136.5 | 2.452 | 2.503 | 135.0 | 2.451 | 2.502 | 135.1 |
|  | (C_5_H_9_O_2_)_2_Sn(dmit) | 2.469 | 2.470 | 126.3 | 2.445 | 2.445 | 125.1 | 2.446 | 2.446 | 125.0 |
|  | (C_3_H_6_NO)_2_Sn(dmit) | 2.511 | 2.534 | 111.7 | 2.471 | 2.521 | 114.6 | 2.433 | 2.508 | 121.5 |

Table S4 - Normalized geometric parameters (bond lengths and bond angles) of the R_2_Sn(dmit) complexes obtained using the min–max scaling method.

|  |  | def2-SVP | | | def2-TZVP | | | def2-QZVP | | |
| --- | --- | --- | --- | --- | --- | --- | --- | --- | --- | --- |
| DFT | Structures | Sn – S_1_ | Sn – S_2_ | C - Sn - C | Sn – S_1_ | Sn – S_2_ | C - Sn - C | Sn – S_1_ | Sn – S_2_ | C - Sn - C |
| PBE | Me_2_Sn(dmit) | 0.030 | 0.000 | 0.265 | 0.000 | 0.000 | 0.154 | 0.000 | 0.000 | 0.145 |
|  | MePhSn(dmit) | 0.000 | 0.048 | 0.218 | 0.038 | 0.030 | 0.108 | 0.031 | 0.030 | 0.093 |
|  | Ph_2_Sn(dmit) | 0.084 | 0.040 | 0.205 | 0.078 | 0.043 | 0.082 | 0.059 | 0.038 | 0.067 |
|  | Et_2_Sn(dmit) | 0.143 | 0.116 | 0.368 | 0.149 | 0.103 | 0.255 | 0.146 | 0.105 | 0.245 |
|  | t-Bu_2_Sn(dmit) | 0.385 | 0.241 | 0.553 | 0.442 | 0.252 | 0.499 | 0.429 | 0.250 | 0.488 |
|  | (C_4_H_7_O_2_)_2_Sn(dmit) | 0.533 | 0.400 | 0.851 | 0.608 | 0.411 | 0.840 | 0.617 | 0.426 | 0.852 |
|  | (C_6_H_11_O)_2_Sn(dmit) | 0.628 | 1.000 | 1.000 | 0.680 | 1.000 | 1.000 | 0.660 | 1.000 | 1.000 |
|  | (C_5_H_9_O_2_)_2_Sn(dmit) | 0.205 | 0.121 | 0.431 | 0.211 | 0.118 | 0.301 | 0.199 | 0.118 | 0.287 |
|  | (C_3_H_6_NO)_2_Sn(dmit) | 1.000 | 0.898 | 0.000 | 1.000 | 0.968 | 0.000 | 1.000 | 0.996 | 0.000 |
| BP86 | Me_2_Sn(dmit) | 0.024 | 0.000 | 0.270 | 0.000 | 0.000 | 0.197 | 0.000 | 0.000 | 0.209 |
|  | MePhSn(dmit) | 0.000 | 0.047 | 0.234 | 0.045 | 0.032 | 0.166 | 0.036 | 0.030 | 0.170 |
|  | Ph_2_Sn(dmit) | 0.076 | 0.037 | 0.217 | 0.079 | 0.044 | 0.140 | 0.066 | 0.038 | 0.146 |
|  | Et_2_Sn(dmit) | 0.159 | 0.112 | 0.386 | 0.181 | 0.105 | 0.314 | 0.179 | 0.103 | 0.321 |
|  | t-Bu_2_Sn(dmit) | 0.413 | 0.261 | 0.587 | 0.510 | 0.266 | 0.557 | 0.506 | 0.264 | 0.557 |
|  | (C_4_H_7_O_2_)_2_Sn(dmit) | 0.548 | 0.410 | 0.870 | 0.678 | 0.425 | 0.868 | 0.698 | 0.432 | 0.883 |
|  | (C_6_H_11_O)_2_Sn(dmit) | 0.619 | 1.000 | 1.000 | 0.739 | 1.000 | 1.000 | 0.730 | 1.000 | 1.000 |
|  | (C_5_H_9_O_2_)_2_Sn(dmit) | 0.185 | 0.114 | 0.386 | 0.213 | 0.112 | 0.295 | 0.204 | 0.108 | 0.299 |
|  | (C_3_H_6_NO)_2_Sn(dmit) | 1.000 | 0.890 | 0.000 | 1.000 | 0.916 | 0.000 | 1.000 | 0.917 | 0.000 |

Table S4 - Continued

|  |  | def2-SVP | | | def2-TZVP | | | def2-QZVP | | |
| --- | --- | --- | --- | --- | --- | --- | --- | --- | --- | --- |
| DFT | Structures | Sn – S_1_ | Sn – S_2_ | C - Sn - C | Sn – S_1_ | Sn – S_2_ | C - Sn - C | Sn – S_1_ | Sn – S_2_ | C - Sn - C |
| M06-L | Me_2_Sn(dmit) | 0.006 | 0.000 | 0.153 | 0.000 | 0.000 | 0.128 | 0.000 | 0.000 | 0.141 |
|  | MePhSn(dmit) | 0.016 | 0.000 | 0.063 | 0.044 | 0.023 | 0.045 | 0.044 | 0.022 | 0.046 |
|  | Ph_2_Sn(dmit) | 0.020 | 0.028 | 0.084 | 0.054 | 0.040 | 0.045 | 0.041 | 0.032 | 0.033 |
|  | Et_2_Sn(dmit) | 0.052 | 0.092 | 0.194 | 0.047 | 0.096 | 0.146 | 0.041 | 0.086 | 0.074 |
|  | t-Bu_2_Sn(dmit) | 0.214 | 0.183 | 0.497 | 0.245 | 0.198 | 0.482 | 0.244 | 0.188 | 0.481 |
|  | (C_4_H_7_O_2_)_2_Sn(dmit) | 0.000 | 0.606 | 0.623 | 0.112 | 0.631 | 0.725 | 0.133 | 0.674 | 0.701 |
|  | (C_6_H_11_O)_2_Sn(dmit) | 0.381 | 1.000 | 1.000 | 0.407 | 0.891 | 1.000 | 0.386 | 0.918 | 1.000 |
|  | (C_5_H_9_O_2_)_2_Sn(dmit) | 0.234 | 0.214 | 0.544 | 0.255 | 0.228 | 0.546 | 0.305 | 0.120 | 0.583 |
|  | (C_3_H_6_NO)_2_Sn(dmit) | 1.000 | 0.979 | 0.000 | 1.000 | 1.000 | 0.000 | 1.000 | 1.000 | 0.000 |
| TPSS | Me_2_Sn(dmit) | 0.0246 | 0.0000 | 0.2464 | 0.0000 | 0.0000 | 0.1984 | 0.000 | 0.000 | 0.200 |
|  | MePhSn(dmit) | 0.0000 | 0.0408 | 0.2018 | 0.0284 | 0.0306 | 0.1597 | 0.025 | 0.030 | 0.157 |
|  | Ph_2_Sn(dmit) | 0.0728 | 0.0371 | 0.1948 | 0.0609 | 0.0412 | 0.1421 | 0.049 | 0.036 | 0.139 |
|  | Et_2_Sn(dmit) | 0.1251 | 0.1081 | 0.3373 | 0.1167 | 0.0930 | 0.2928 | 0.111 | 0.092 | 0.294 |
|  | t-Bu_2_Sn(dmit) | 0.3112 | 0.2243 | 0.5373 | 0.3349 | 0.2382 | 0.5320 | 0.323 | 0.232 | 0.518 |
|  | (C_4_H_7_O_2_)_2_Sn(dmit) | 0.4876 | 0.4128 | 0.8262 | 0.5429 | 0.4486 | 0.8522 | 0.548 | 0.459 | 0.860 |
|  | (C_6_H_11_O)_2_Sn(dmit) | 0.5458 | 1.0000 | 1.0000 | 0.5677 | 1.0000 | 1.0000 | 0.543 | 1.000 | 1.000 |
|  | (C_5_H_9_O_2_)_2_Sn(dmit) | 0.1577 | 0.1079 | 0.3727 | 0.1578 | 0.1096 | 0.3115 | 0.150 | 0.105 | 0.302 |
|  | (C_3_H_6_NO)_2_Sn(dmit) | 1.0000 | 0.8135 | 0.0000 | 1.0000 | 0.9461 | 0.0000 | 1.000 | 0.961 | 0.000 |

Table S4 - Continued

|  |  | def2-SVP | | | def2-TZVP | | | def2-QZVP | | |
| --- | --- | --- | --- | --- | --- | --- | --- | --- | --- | --- |
| DFT | Structures | Sn – S_1_ | Sn – S_2_ | C - Sn - C | Sn – S_1_ | Sn – S_2_ | C - Sn - C | Sn – S_1_ | Sn – S_2_ | C - Sn - C |
| r^2^SCAN | Me_2_Sn(dmit) | 0.000 | 0.000 | 0.236 | 0.000 | 0.000 | 0.200 | 0.000 | 0.000 | 0.205 |
|  | MePhSn(dmit) | 0.000 | 0.005 | 0.182 | 0.020 | 0.016 | 0.145 | 0.014 | 0.014 | 0.143 |
|  | Ph_2_Sn(dmit) | 0.018 | 0.014 | 0.138 | 0.035 | 0.027 | 0.094 | 0.026 | 0.026 | 0.096 |
|  | Et_2_Sn(dmit) | 0.080 | 0.099 | 0.334 | 0.084 | 0.090 | 0.246 | 0.078 | 0.078 | 0.242 |
|  | t-Bu_2_Sn(dmit) | 0.226 | 0.196 | 0.524 | 0.261 | 0.206 | 0.486 | 0.250 | 0.194 | 0.485 |
|  | (C_4_H_7_O_2_)_2_Sn(dmit) | 0.071 | 0.672 | 0.730 | 0.080 | 0.669 | 0.651 | 0.075 | 0.666 | 0.651 |
|  | (C_6_H_11_O)_2_Sn(dmit) | 0.419 | 1.000 | 1.000 | 0.434 | 1.000 | 1.000 | 0.421 | 1.000 | 1.000 |
|  | (C_5_H_9_O_2_)_2_Sn(dmit) | 0.230 | 0.192 | 0.593 | 0.164 | 0.126 | 0.445 | 0.186 | 0.140 | 0.503 |
|  | (C_3_H_6_NO)_2_Sn(dmit) | 1.000 | 0.877 | 0.000 | 1.000 | 0.974 | 0.000 | 1.000 | 0.983 | 0.000 |
| B3LYP | Me_2_Sn(dmit) | 0.0000 | 0.0000 | 0.2981 | 0.0000 | 0.0000 | 0.0967 | 0.000 | 0.000 | 0.087 |
|  | MePhSn(dmit) | 0.0040 | 0.0469 | 0.2267 | 0.1180 | 0.0395 | 0.0144 | 0.047 | 0.021 | 0.027 |
|  | Ph_2_Sn(dmit) | 0.0561 | 0.0397 | 0.2013 | 0.0903 | 0.0365 | 0.0000 | 0.076 | 0.023 | 0.000 |
|  | Et_2_Sn(dmit) | 0.1620 | 0.1205 | 0.3859 | 0.2794 | 0.1119 | 0.1508 | 0.250 | 0.100 | 0.216 |
|  | t-Bu_2_Sn(dmit) | 0.4500 | 0.2634 | 0.5544 | 0.7858 | 0.2713 | 0.4254 | 0.739 | 0.246 | 0.454 |
|  | (C_4_H_7_O_2_)_2_Sn(dmit) | 0.2721 | 0.7016 | 0.9066 | 0.2468 | 0.6828 | 0.6785 | 0.348 | 0.727 | 0.787 |
|  | (C_6_H_11_O)_2_Sn(dmit) | 0.6396 | 1.0000 | 1.0000 | 1.0000 | 0.9674 | 1.0000 | 1.000 | 1.000 | 1.000 |
|  | (C_5_H_9_O_2_)_2_Sn(dmit) | 0.2253 | 0.1391 | 0.4114 | 0.3056 | 0.1339 | 0.0738 | 0.312 | 0.102 | 0.147 |
|  | (C_3_H_6_NO)_2_Sn(dmit) | 1.0000 | 0.8964 | 0.0000 | 0.3876 | 1.0000 | 0.2964 | 0.301 | 0.945 | 0.323 |

Table S4 - Continued

|  |  | def2-SVP | | | def2-TZVP | | | def2-QZVP | | |
| --- | --- | --- | --- | --- | --- | --- | --- | --- | --- | --- |
| DFT | Structures | Sn – S_1_ | Sn – S_2_ | C - Sn - C | Sn – S_1_ | Sn – S_2_ | C - Sn - C | Sn – S_1_ | Sn – S_2_ | C - Sn - C |
| PBE0 | Me_2_Sn(dmit) | 0.038 | 0.000 | 0.249 | 0.000 | 0.000 | 0.153 | 0.000 | 0.000 | 0.163 |
|  | MePhSn(dmit) | 0.000 | 0.028 | 0.195 | 0.017 | 0.013 | 0.100 | 0.016 | 0.015 | 0.104 |
|  | Ph_2_Sn(dmit) | 0.040 | 0.007 | 0.175 | 0.027 | 0.016 | 0.066 | 0.020 | 0.011 | 0.070 |
|  | Et_2_Sn(dmit) | 0.115 | 0.097 | 0.346 | 0.119 | 0.084 | 0.236 | 0.123 | 0.083 | 0.256 |
|  | t-Bu_2_Sn(dmit) | 0.295 | 0.196 | 0.537 | 0.404 | 0.201 | 0.475 | 0.375 | 0.199 | 0.483 |
|  | (C_4_H_7_O_2_)_2_Sn(dmit) | 0.227 | 0.717 | 0.886 | 0.197 | 0.702 | 0.741 | 0.182 | 0.707 | 0.740 |
|  | (C_6_H_11_O)_2_Sn(dmit) | 0.487 | 1.000 | 1.000 | 0.642 | 0.964 | 1.000 | 0.593 | 0.970 | 1.000 |
|  | (C_5_H_9_O_2_)_2_Sn(dmit) | 0.175 | 0.104 | 0.412 | 0.204 | 0.100 | 0.296 | 0.193 | 0.099 | 0.298 |
|  | (C_3_H_6_NO)_2_Sn(dmit) | 1.000 | 0.876 | 0.000 | 1.000 | 1.000 | 0.000 | 1.000 | 1.000 | 0.000 |
| CAM-B3LYP | Me_2_Sn(dmit) | 0.0082 | 0.0000 | 0.3293 | 0.0000 | 0.0000 | 0.1349 | 0.000 | 0.000 | 0.104 |
|  | MePhSn(dmit) | 0.0000 | 0.0470 | 0.2430 | 0.0331 | 0.0370 | 0.0000 | 0.013 | 0.008 | 0.027 |
|  | Ph_2_Sn(dmit) | 0.0180 | 0.0240 | 0.2118 | 0.0461 | 0.0181 | 0.0008 | 0.009 | 0.004 | 0.000 |
|  | Et_2_Sn(dmit) | 0.1037 | 0.1059 | 0.3953 | 0.2556 | 0.0992 | 0.1219 | 0.194 | 0.085 | 0.212 |
|  | t-Bu_2_Sn(dmit) | 0.3114 | 0.1896 | 0.5508 | 0.7607 | 0.2211 | 0.4148 | 0.656 | 0.188 | 0.407 |
|  | (C_4_H_7_O_2_)_2_Sn(dmit) | 0.2551 | 0.7494 | 0.9079 | 0.2295 | 0.7354 | 0.6484 | 0.242 | 0.742 | 0.706 |
|  | (C_6_H_11_O)_2_Sn(dmit) | 0.4871 | 1.0000 | 1.0000 | 1.0000 | 0.9896 | 1.0000 | 1.000 | 1.000 | 1.000 |
|  | (C_5_H_9_O_2_)_2_Sn(dmit) | 0.1913 | 0.1254 | 0.3446 | 0.4590 | 0.1356 | 0.1694 | 0.335 | 0.095 | 0.206 |
|  | (C_3_H_6_NO)_2_Sn(dmit) | 1.0000 | 0.9059 | 0.0000 | 0.3003 | 1.0000 | 0.3755 | 0.286 | 0.977 | 0.370 |

Table S4 - Continued

|  |  | def2-SVP | | | def2-TZVP | | | def2-QZVP | | |
| --- | --- | --- | --- | --- | --- | --- | --- | --- | --- | --- |
| DFT | Structures | Sn – S_1_ | Sn – S_2_ | C - Sn - C | Sn – S_1_ | Sn – S_2_ | C - Sn - C | Sn – S_1_ | Sn – S_2_ | C - Sn - C |
| M06-2X | Me_2_Sn(dmit) | 0.0000 | 0.0000 | 0.3614 | 0.0000 | 0.0000 | 0.4245 | 0.008 | 0.013 | 0.226 |
|  | MePhSn(dmit) | 0.0100 | 0.0144 | 0.1972 | 0.0168 | 0.0106 | 0.2460 | 0.000 | 0.000 | 0.122 |
|  | Ph_2_Sn(dmit) | 0.0066 | 0.0082 | 0.1521 | 0.0266 | 0.0129 | 0.2235 | 0.005 | 0.006 | 0.095 |
|  | Et_2_Sn(dmit) | 0.0629 | 0.0928 | 0.3484 | 0.0768 | 0.1205 | 0.3145 | 0.041 | 0.091 | 0.196 |
|  | t-Bu_2_Sn(dmit) | 0.2154 | 0.1926 | 0.5621 | 0.2275 | 0.2618 | 0.5019 | 0.201 | 0.189 | 0.424 |
|  | (C_4_H_7_O_2_)_2_Sn(dmit) | 0.1708 | 0.6922 | 0.7689 | 0.0402 | 0.7512 | 0.7008 | 0.705 | 0.667 | 0.655 |
|  | (C_6_H_11_O)_2_Sn(dmit) | 0.4159 | 1.0000 | 1.0000 | 0.3540 | 0.9509 | 1.0000 | 1.000 | 0.949 | 1.000 |
|  | (C_5_H_9_O_2_)_2_Sn(dmit) | 0.3126 | 0.2350 | 0.6338 | 0.3108 | 0.2346 | 0.5392 | 0.251 | 0.237 | 0.588 |
|  | (C_3_H_6_NO)_2_Sn(dmit) | 1.0000 | 0.8933 | 0.0000 | 1.0000 | 1.0000 | 0.0000 | 0.841 | 1.000 | 0.000 |
| ωB97X-D3 | Me_2_Sn(dmit) | 0.0619 | 0.0082 | 0.2084 | 0.0015 | 0.0115 | 0.1991 | 0.048 | 0.024 | 0.158 |
|  | MePhSn(dmit) | 0.0363 | 0.0181 | 0.1363 | 0.0316 | 0.0155 | 0.0610 | 0.036 | 0.017 | 0.082 |
|  | Ph_2_Sn(dmit) | 0.0000 | 0.0000 | 0.1547 | 0.0000 | 0.0000 | 0.0000 | 0.000 | 0.000 | 0.000 |
|  | Et_2_Sn(dmit) | 0.0895 | 0.0824 | 0.2884 | 0.1959 | 0.0914 | 0.1870 | 0.085 | 0.085 | 0.162 |
|  | t-Bu_2_Sn(dmit) | 0.1810 | 0.1571 | 0.5377 | 0.7390 | 0.1971 | 0.5143 | 0.259 | 0.152 | 0.415 |
|  | (C_4_H_7_O_2_)_2_Sn(dmit) | 0.0684 | 0.5972 | 0.6964 | 0.0888 | 0.6337 | 0.5860 | 0.071 | 0.569 | 0.616 |
|  | (C_6_H_11_O)_2_Sn(dmit) | 0.3695 | 0.8239 | 1.0000 | 1.0000 | 0.8924 | 1.0000 | 0.444 | 0.824 | 1.000 |
|  | (C_5_H_9_O_2_)_2_Sn(dmit) | 0.3232 | 0.2019 | 0.6079 | 0.7918 | 0.2262 | 0.5513 | 0.364 | 0.195 | 0.521 |
|  | (C_3_H_6_NO)_2_Sn(dmit) | 1.0000 | 1.0000 | 0.0000 | 0.2896 | 1.0000 | 0.4362 | 1.000 | 1.000 | 0.047 |

Table S4 - Continued

|  |  | def2-SVP | | | def2-TZVP | | | def2-QZVP | | |
| --- | --- | --- | --- | --- | --- | --- | --- | --- | --- | --- |
| DFT | Structures | Sn – S_1_ | Sn – S_2_ | C - Sn - C | Sn – S_1_ | Sn – S_2_ | C - Sn - C | Sn – S_1_ | Sn – S_2_ | C - Sn - C |
| ωB97M-V | Me_2_Sn(dmit) | 0.0214 | 0.0126 | 0.2365 | 0.0131 | 0.0000 | 0.1415 | 0.048 | 0.022 | 0.158 |
|  | MePhSn(dmit) | 0.0000 | 0.0333 | 0.1272 | 0.4006 | 0.2384 | 0.0608 | 0.036 | 0.016 | 0.082 |
|  | Ph_2_Sn(dmit) | 0.0000 | 0.0000 | 0.1180 | 0.0000 | 0.0065 | 0.0000 | 0.000 | 0.000 | 0.000 |
|  | Et_2_Sn(dmit) | 0.0503 | 0.1090 | 0.2933 | 0.0761 | 0.0765 | 0.0999 | 0.087 | 0.084 | 0.163 |
|  | t-Bu_2_Sn(dmit) | 0.1871 | 0.1603 | 0.5111 | 0.2499 | 0.1604 | 0.3761 | 0.256 | 0.152 | 0.416 |
|  | (C_4_H_7_O_2_)_2_Sn(dmit) | 0.1012 | 0.6488 | 0.8072 | 0.0797 | 0.5640 | 0.6189 | 0.071 | 0.571 | 0.617 |
|  | (C_6_H_11_O)_2_Sn(dmit) | 0.3379 | 0.8498 | 1.0000 | 0.4071 | 0.7551 | 1.0000 | 0.444 | 0.825 | 1.000 |
|  | (C_5_H_9_O_2_)_2_Sn(dmit) | 0.2956 | 0.2144 | 0.5760 | 0.3621 | 0.1859 | 0.5334 | 0.361 | 0.195 | 0.520 |
|  | (C_3_H_6_NO)_2_Sn(dmit) | 1.0000 | 1.0000 | 0.0000 | 1.0000 | 1.0000 | 0.0072 | 1.000 | 1.000 | 0.046 |
| B2PLYP-D3 | Me_2_Sn(dmit) | 0.024 | 0.000 | 0.177 | 0.575 | 0.233 | 0.149 | 0.013 | 0.004 | 0.146 |
|  | MePhSn(dmit) | 0.000 | 0.010 | 0.113 | 0.550 | 0.244 | 0.077 | 0.022 | 0.007 | 0.071 |
|  | Ph_2_Sn(dmit) | 0.008 | 0.000 | 0.043 | 0.550 | 0.233 | 0.000 | 0.000 | 0.000 | 0.000 |
|  | Et_2_Sn(dmit) | 0.075 | 0.084 | 0.241 | 0.025 | 0.000 | 0.203 | 0.181 | 0.086 | 0.217 |
|  | t-Bu_2_Sn(dmit) | 0.208 | 0.144 | 0.494 | 0.850 | 0.360 | 0.500 | 0.591 | 0.186 | 0.467 |
|  | (C_4_H_7_O_2_)_2_Sn(dmit) | 0.035 | 0.565 | 0.635 | 0.000 | 0.512 | 0.505 | 0.095 | 0.626 | 0.588 |
|  | (C_6_H_11_O)_2_Sn(dmit) | 0.372 | 0.855 | 1.000 | 0.525 | 0.791 | 1.000 | 1.000 | 0.928 | 1.000 |
|  | (C_5_H_9_O_2_)_2_Sn(dmit) | 0.280 | 0.190 | 0.590 | 0.350 | 0.116 | 0.554 | 0.793 | 0.229 | 0.559 |
|  | (C_3_H_6_NO)_2_Sn(dmit) | 1.000 | 1.000 | 0.000 | 1.000 | 1.000 | 0.081 | 0.224 | 1.000 | 0.408 |

Table S5 - Statistical parameters used to construct the box plots for the deviations in the normalized geometric parameters. The deviations were calculated as the difference between the calculated and experimental normalized values. The table reports the minimum, first quartile (Q1), median, third quartile (Q3), maximum values, and the box range (interquartile range, IQR).

| Theory level | Box range | Min | Q1 | Median | Q3 | Max |
| --- | --- | --- | --- | --- | --- | --- |
| def2-SVP | | | | | | |
| PBE | 0.5580 | -0.9450 | -0.4720 | -0.1310 | 0.0860 | 0.5070 |
| BP86 | 0.5350 | -0.9490 | -0.4530 | -0.1250 | 0.0820 | 0.5270 |
| M06-L | 0.5630 | -0.9570 | -0.5110 | -0.2550 | 0.0520 | 0.4330 |
| TPSS | 0.5802 | -0.9485 | -0.5023 | -0.1506 | 0.0778 | 0.4828 |
| r^2^SCAN | 0.4960 | -0.9720 | -0.4280 | -0.2425 | 0.0680 | 0.4980 |
| B3LYP | 0.5440 | -0.9459 | -0.4538 | -0.1526 | 0.0910 | 0.5640 |
| PBE0 | 0.5610 | -0.9790 | -0.4940 | -0.2135 | 0.0670 | 0.5440 |
| CAM-B3LYP | 0.5200 | -0.9616 | -0.4444 | -0.2242 | 0.0757 | 0.5762 |
| M06-2X | 0.4379 | -0.9774 | -0.3754 | -0.2448 | 0.0625 | 0.5190 |
| ωB97X-D3 | 0.4595 | -0.9856 | -0.4223 | -0.2417 | 0.0372 | 0.4241 |
| ωB97M-V | 0.5036 | -0.9856 | -0.4508 | -0.2663 | 0.0528 | 0.4756 |
| B2PLYP-D3 | 0.5410 | -0.9860 | -0.4870 | -0.2590 | 0.0540 | 0.3920 |
| def2-TZVP | | | | | | |
| PBE | 0.6830 | -0.9420 | -0.5850 | -0.1400 | 0.0980 | 0.4960 |
| BP86 | 0.6310 | -0.9420 | -0.5260 | -0.0970 | 0.1050 | 0.5240 |
| M06-L | 0.5810 | -0.9450 | -0.5360 | -0.2675 | 0.0450 | 0.4580 |
| TPSS | 0.6112 | -0.9444 | -0.5469 | -0.1829 | 0.0643 | 0.5089 |
| r^2^SCAN | 0.5400 | -0.9590 | -0.4810 | -0.2315 | 0.0590 | 0.4960 |
| B3LYP | 0.6940 | -0.9490 | -0.5930 | -0.1900 | 0.1010 | 0.6530 |
| PBE0 | 0.6580 | -0.9700 | -0.6040 | -0.2185 | 0.0540 | 0.5290 |
| CAM-B3LYP | 0.7091 | -0.9675 | -0.6309 | -0.1914 | 0.0783 | 0.6354 |
| M06-2X | 0.5236 | -0.9727 | -0.4468 | -0.2032 | 0.0768 | 0.5780 |
| ωB97X-D3 | 0.6426 | -0.9856 | -0.5118 | -0.1839 | 0.1307 | 0.6193 |
| ωB97M-V | 0.5972 | -0.9791 | -0.5826 | -0.2629 | 0.0146 | 0.3908 |
| B2PLYP-D3 | 0.6460 | -0.8650 | -0.5150 | -0.1580 | 0.1310 | 0.7250 |
| def2-QZVP | | | | | | |
| PBE | 0.6880 | -0.9470 | -0.5950 | -0.1515 | 0.0930 | 0.5090 |
| BP86 | 0.6220 | -0.9480 | -0.5190 | -0.0995 | 0.1030 | 0.5390 |
| M06-L | 0.6500 | -0.9530 | -0.6090 | -0.2765 | 0.0410 | 0.5010 |
| TPSS | 0.6080 | -0.9500 | -0.5460 | -0.1880 | 0.0620 | 0.5170 |
| r^2^SCAN | 0.5160 | -0.9650 | -0.4590 | -0.2395 | 0.0570 | 0.4920 |
| B3LYP | 0.7090 | -0.9620 | -0.6240 | -0.1630 | 0.0850 | 0.6140 |
| PBE0 | 0.6370 | -0.9750 | -0.5840 | -0.2165 | 0.0530 | 0.5340 |
| CAM-B3LYP | 0.6810 | -0.9820 | -0.6270 | -0.2495 | 0.0540 | 0.5690 |
| M06-2X | 0.5480 | -0.9790 | -0.4870 | -0.1940 | 0.0610 | 0.4940 |
| ωB97X-D3 | 0.5660 | -0.9860 | -0.5200 | -0.2525 | 0.0460 | 0.3960 |
| ωB97M-V | 0.5670 | -0.9860 | -0.5200 | -0.2535 | 0.0470 | 0.3970 |
| B2PLYP-D3 | 0.6160 | -0.9860 | -0.5180 | -0.2010 | 0.0980 | 0.4650 |

Table S6 – Experimental and calculated infrared (IR) vibrational frequencies (cm^−1^) after linear scaling corrections for the R_2_Sn(dmit) complexes obtained at different levels of theory.

| BOND | | EXP | PBE | BP86 | M06-L | TPSS | r^2^SCAN | B3LYP | PBE0 | CAM-B3LYP | M06-2X | ωB97X-D3 | ωB97M-V | B2PLYP-D3 | |
| --- | --- | --- | --- | --- | --- | --- | --- | --- | --- | --- | --- | --- | --- | --- | --- |
|  | def2-SVP | | | | | | | | | | | | | |  |
| Sn-S | | 327 | 321 | 346 | 383 | 360 | 380 | 367 | 407 | 416 | 407 | 434 | 422 | 367 | |
| C-S | | 463 | 449 | 456 | 499 | 469 | 494 | 481 | 522 | 526 | 517 | 543 | 526 | 498 | |
| C-S | | 895 | 868 | 864 | 934 | 890 | 926 | 906 | 983 | 985 | 982 | 1020 | 991 | 943 | |
| C=S | | 1026 | 1075 | 1105 | 1188 | 1187 | 1176 | 1157 | 1224 | 1232 | 1232 | 1257 | 1237 | 1185 | |
| C=C | | 1447 | 1461 | 1487 | 1585 | 1530 | 1568 | 1591 | 1657 | 1699 | 1693 | 1714 | 1706 | 1597 | |
| C-H | | 2869 | 2981 | 2987 | 3149 | 3077 | 3128 | 3142 | 3204 | 3226 | 3220 | 3232 | 3211 | 3145 | |
|  | def2-TZVP | | | | | | | | | | | | | |  |
| Sn-S | | 327 | 344 | 335 | 367 | 356 | 373 | 356 | 399 | 373 | 397 | 425 | 434 | 391 | |
| C-S | | 463 | 453 | 444 | 478 | 513 | 483 | 468 | 510 | 375 | 507 | 527 | 593 | 500 | |
| C-S | | 895 | 873 | 855 | 923 | 884 | 917 | 894 | 975 | 949 | 974 | 1003 | 998 | 947 | |
| C=S | | 1026 | 1084 | 1068 | 1139 | 1093 | 1138 | 1115 | 1185 | 1170 | 1189 | 1212 | 1204 | 1186 | |
| C=C | | 1447 | 1470 | 1458 | 1555 | 1495 | 1536 | 1559 | 1624 | 1659 | 1656 | 1686 | 1685 | 1602 | |
| C-H | | 2869 | 2989 | 2976 | 3161 | 3044 | 3106 | 3127 | 3175 | 3227 | 3214 | 3219 | 3198 | 3200 | |
|  | def2-QZVP | | | | | | | | | | | | | |  |
| Sn-S | | 327 | 342 | 333 | 361 | 341 | 372 | 356 | 397 | 404 | 397 | 410 | 414 | - | |
| C-S | | 463 | 454 | 444 | 474 | 449 | 484 | 468 | 509 | 510 | 504 | 523 | 512 | - | |
| C-S | | 895 | 872 | 853 | 926 | 872 | 919 | 893 | 974 | 968 | 966 | 982 | 981 | - | |
| C=S | | 1026 | 1078 | 1063 | 1147 | 1081 | 1135 | 1111 | 1180 | 1180 | 1177 | 1180 | 1184 | - | |
| C=C | | 1447 | 1463 | 1451 | 1557 | 1484 | 1531 | 1554 | 1618 | 1662 | 1647 | 1671 | 1676 | - | |
| C-H | | 2869 | 2978 | 2965 | 3246 | 3042 | 3094 | 3120 | 3168 | 3199 | 3206 | 3196 | 3193 | - | |

Table S7 – Compounds in the dataset with the solvent used in the experimental measurement, the experimental ^119^Sn NMR chemical shift δexp, the temperature, and the reference.

| Structure | Solvent | δexp (ppm) | T (°C) | REF |
| --- | --- | --- | --- | --- |
| Me_2_Sn(dmit) | acetone | 135.2 | NR | [4] |
| MePhSn(dmit) | acetone | 75.2 | NR | [4] |
| Ph_2_Sn(dmit) | acetone | -24.8 | NR | [4] |
| Et_2_Sn(dmit) | acetone | 165.6 | NR | [4] |
| Bu_2_Sn(dmit) | acetone | 161.5 | NR | [4] |
| (C_4_H_7_O_2_)_2_Sn(dmit) | acetone | 84.4 | NR | [5] |
| (C_5_H_9_O_2_)_2_Sn(dmit) | dichloromethane | 84.2 | NR | [6] |
| C_26_H_24_SSn | chloroform | -115.5 | NR | [1] |
| C_6_H_12_N_2_S_3_Sn | chloroform | -270.1 | 20 | [2] |
| C_16_H_25_N_3_S_3_Sn | chloroform | -427.3 | 20 | [2] |
| C_21_H_35_N_3_S_6_Sn | dichloromethane | -813 | 25 | [3] |

* NR = not reported.

Table S8 – Calculated ^119^Sn nuclear magnetic shielding values (ppm) obtained with different density functionals with DKH2 [SARC-DKH-TZVP(Sn)/DKH-def2-TZVP(other atoms)], ZORA [SARC-ZORA-TZVP(Sn)/ZORA-def2-TZVP(other atoms)], and X2C [X2C-TZVPall-s] methods.

| SARC-DKH-TZVP | | | | | | | | | | | | |
| --- | --- | --- | --- | --- | --- | --- | --- | --- | --- | --- | --- | --- |
| Structure | PBE | BP86 | M06-L | TPSS | r^2^SCAN | B3LYP | PBE0 | CAM-B3LYP | M06-2X | ωB97X-D3 | ωB97M-V | B2PLYP-D3 |
| Me_2_Sn(dmit) | 3325.1 | 3303.9 | 3497.7 | 3386.4 | 3459.0 | 3354.1 | 3413.2 | 3387.7 | 3466.7 | 3436.0 | 3491.6 | 2168.0 |
| MePhSn(dmit) | 3385.2 | 3365.9 | 3554.8 | 3445.5 | 3514.0 | 3408.8 | 3466.5 | 3437.9 | 3517.5 | 3485.2 | 3539.6 | 2269.5 |
| Ph_2_Sn(dmit) | 3449.4 | 3431.0 | 3611.5 | 3507.3 | 3572.9 | 3466.0 | 3523.4 | 3491.2 | 3570.1 | 3537.6 | 3591.3 | 2378.3 |
| Et_2_Sn(dmit) | 3303.6 | 3282.1 | 3481.4 | 3367.0 | 3438.5 | 3330.1 | 3391.5 | 3362.4 | 3442.4 | 3412.1 | 3468.0 | 2175.1 |
| Bu_2_Sn(dmit) | 3320.9 | 3300.0 | 3504.4 | 3384.0 | 3462.6 | 3345.0 | 3405.8 | 3378.6 | 3450.3 | 3426.8 | 3481.6 | 2212.6 |
| (C_4_H_7_O_2_)_2_Sn(dmit) | 3367.9 | 3347.4 | 3549.1 | 3434.8 | 3504.9 | 3390.1 | 3450.4 | 3421.3 | 3490.3 | 3469.2 | 3527.4 | 2283.4 |
| (C_5_H_9_O_2_)_2_Sn(dmit) | 3411.9 | 3392.0 | 3582.5 | 3475.1 | 3537.9 | 3435.7 | 3495.2 | 3467.6 | 3551.6 | 3514.1 | 3574.0 | 2390.3 |
| C_26_H_24_SSn | 3638.7 | 3623.7 | 3775.0 | 3690.0 | 3738.0 | 3648.2 | 3698.8 | 3667.0 | 3738.4 | 3704.3 | 3759.2 | 2599.3 |
| C_6_H_12_N_2_S_3_Sn | 3757.1 | 3741.3 | 3890.1 | 3797.3 | 3857.5 | 3765.9 | 3813.8 | 3782.5 | 3854.6 | 3818.9 | 3870.8 | 2741.2 |
| C_16_H_25_N_3_S_3_Sn | 3731.3 | 3713.0 | 3867.9 | 3786.2 | 3832.3 | 3739.4 | 3799.6 | 3763.0 | 3842.2 | 3804.0 | 3861.7 | 2799.1 |
| C_21_H_35_N_3_S_6_Sn | 4154.8 | 4139.0 | 4242.6 | 4189.8 | 4227.1 | 4149.6 | 4208.3 | 4174.9 | 4231.5 | 4210.9 | 4260.8 | 3387.4 |

Table S8 – Continued

| SARC-ZORA-TZVP | | | | | | | | | | | | |
| --- | --- | --- | --- | --- | --- | --- | --- | --- | --- | --- | --- | --- |
| Structure | PBE | BP86 | M06-L | TPSS | r^2^SCAN | B3LYP | PBE0 | CAM-B3LYP | M06-2X | ωB97X-D3 | ωB97M-V | B2PLYP-D3 |
| Me_2_Sn(dmit) | 3383.3 | 3362.6 | 3552.3 | 3442.9 | 3514.2 | 3411.7 | 3469.4 | 3444.5 | 3522.3 | 3491.5 | 3545.8 | 2236.7 |
| MePhSn(dmit) | 3441.5 | 3422.7 | 3607.6 | 3500.2 | 3567.5 | 3464.6 | 3520.9 | 3492.9 | 3571.5 | 3539.0 | 3592.2 | 2335.9 |
| Ph_2_Sn(dmit) | 3503.7 | 3485.8 | 3662.5 | 3559.9 | 3624.6 | 3520.0 | 3576.1 | 3544.5 | 3622.3 | 3589.7 | 3642.2 | 2442.1 |
| Et_2_Sn(dmit) | 3362.1 | 3341.2 | 3536.3 | 3423.8 | 3494.1 | 3388.2 | 3448.0 | 3419.6 | 3498.5 | 3468.0 | 3522.6 | 2243.9 |
| Bu_2_Sn(dmit) | 3378.6 | 3358.2 | 3558.0 | 3439.8 | 3516.7 | 3402.3 | 3461.6 | 3435.1 | 3506.3 | 3481.9 | 3535.7 | 2280.6 |
| (C_4_H_7_O_2_)_2_Sn(dmit) | 3424.0 | 3404.1 | 3601.3 | 3488.9 | 3557.7 | 3445.9 | 3504.7 | 3476.3 | 3545.0 | 3522.9 | 3575.4 | 2349.0 |
| (C_5_H_9_O_2_)_2_Sn(dmit) | 3466.6 | 3447.2 | 3633.8 | 3528.0 | 3589.9 | 3490.0 | 3548.0 | 3521.0 | 3603.9 | 3566.3 | 3624.7 | 2453.5 |
| C_26_H_24_SSn | 3688.9 | 3674.2 | 3822.3 | 3738.7 | 3786.0 | 3698.2 | 3747.6 | 3716.5 | 3786.9 | 3752.8 | 3806.5 | 2659.2 |
| C_6_H_12_N_2_S_3_Sn | 3804.8 | 3789.4 | 3935.2 | 3844.0 | 3903.1 | 3813.6 | 3860.3 | 3829.5 | 3900.6 | 3865.0 | 3915.7 | 2798.2 |
| C_16_H_25_N_3_S_3_Sn | 3778.8 | 3760.8 | 3912.8 | 3832.1 | 3877.7 | 3786.8 | 3845.5 | 3809.7 | 3887.8 | 3849.7 | 3905.9 | 2854.2 |
| C_21_H_35_N_3_S_6_Sn | 4192.2 | 4176.8 | 4278.7 | 4226.2 | 4263.1 | 4187.2 | 4244.4 | 4211.7 | 4267.7 | 4246.8 | 4295.4 | 3430.3 |

Table S8 – Continued

| X2C-TZVPall-s | | | | | | | | | | | | |
| --- | --- | --- | --- | --- | --- | --- | --- | --- | --- | --- | --- | --- |
| Structure | PBE | BP86 | M06-L | TPSS | r^2^SCAN | B3LYP | PBE0 | CAM-B3LYP | M06-2X | ωB97X-D3 | ωB97M-V | B2PLYP-D3 |
| Me_2_Sn(dmit) | 2919.4 | 2893.9 | 3146.4 | 3002.9 | 3078.5 | 2958.6 | 3022.0 | 2997.3 | 3072.0 | 3035.8 | 3092.8 | 2238.8 |
| MePhSn(dmit) | 2974.0 | 2949.2 | 3237.7 | 3070.4 | 3168.2 | 3008.5 | 3072.4 | 3043.8 | 3097.8 | 3083.2 | 3144.7 | 2366.8 |
| Ph_2_Sn(dmit) | 3033.4 | 3009.2 | 3249.9 | 3113.9 | 3183.5 | 3062.0 | 3125.4 | 3094.1 | 3172.0 | 3133.2 | 3187.3 | 2484.1 |
| Et_2_Sn(dmit) | 2918.9 | 2893.1 | 3179.7 | 3014.1 | 3109.2 | 2955.8 | 3020.6 | 2993.5 | 3049.6 | 3033.3 | 3093.2 | 2247.6 |
| Bu_2_Sn(dmit) | 2911.7 | 2885.1 | 3166.2 | 3001.4 | 3091.6 | 2946.0 | 3013.6 | 2985.9 | 3059.0 | 3025.4 | 3091.9 | 2297.6 |
| (C_4_H_7_O_2_)_2_Sn(dmit) | 2983.3 | 2958.4 | 3240.1 | 3076.8 | 3161.3 | 3016.3 | 3081.9 | 3052.8 | 3124.3 | 3093.2 | 3157.7 | 2367.2 |
| (C_5_H_9_O_2_)_2_Sn(dmit) | 3044.9 | 3020.3 | 3273.6 | 3130.9 | 3197.1 | 3080.7 | 3144.9 | 3118.0 | 3199.5 | 3155.4 | 3216.6 | 2467.4 |
| C_26_H_24_SSn | 3180.7 | 3160.7 | 3370.5 | 3249.2 | 3302.8 | 3199.6 | 3253.2 | 3222.8 | 3296.1 | 3252.2 | 3308.5 | 2711.1 |
| C_6_H_12_N_2_S_3_Sn | 3380.7 | 3362.1 | 3560.7 | 3437.8 | 3503.0 | 3397.8 | 3448.1 | 3417.5 | 3486.3 | 3446.1 | 3499.0 | 2826.5 |
| C_16_H_25_N_3_S_3_Sn | 3443.7 | 3420.8 | 3634.9 | 3517.9 | 3569.7 | 3461.7 | 3526.6 | 3489.5 | 3570.7 | 3524.2 | 3582.4 | 2898.5 |
| C_21_H_35_N_3_S_6_Sn | 3827.0 | 3806.6 | 3953.5 | 3877.4 | 3913.0 | 3819.9 | 3882.3 | 3845.1 | 3897.7 | 3877.0 | 3927.8 | 3507.9 |

Table S9 – Calculated ^119^Sn nuclear shielding (ppm) for the tin atom in tetramethyltin (SnMe_4_) with different density functionals with DKH2 [SARC-DKH-TZVP(Sn)/DKH-def2-TZVP(other atoms)], ZORA [SARC-ZORA-TZVP(Sn)/ZORA-def2-TZVP(other atoms)], and X2C [X2C-TZVPall-s] methods.

| Solvent | PBE | BP86 | M06-L | TPSS | r^2^SCAN | B3LYP | PBE0 | CAM-B3LYP | M06-2X | ωB97X-D3 | ωB97M-V | B2PLYP-D3 |
| --- | --- | --- | --- | --- | --- | --- | --- | --- | --- | --- | --- | --- |
| SARC-DKH-TZVP | | | | | | | | | | | | |
| Acetone | 3620.2 | 3606.9 | 3747.1 | 3662.1 | 3717.4 | 3641.6 | 3686.6 | 3666.9 | 3745.4 | 3705.8 | 3756.5 | 2454.2 |
| Dichloromethane | 3620.4 | 3607.0 | 3747.3 | 3662.2 | 3717.5 | 3641.7 | 3686.6 | 3667.0 | 3745.5 | 3705.9 | 3756.6 | 2454.0 |
| Chloroform | 3620.6 | 3607.2 | 3747.6 | 3662.4 | 3717.5 | 3641.9 | 3686.8 | 3667.1 | 3745.7 | 3706.0 | 3756.7 | 2453.8 |
| SARC-ZORA-TZVP | | | | | | | | | | | | |
| Acetone | 3671.5 | 3658.4 | 3795.7 | 3712.3 | 3766.7 | 3692.6 | 3736.4 | 3717.3 | 3794.4 | 3755.1 | 3804.6 | 2516.6 |
| Dichloromethane | 3671.6 | 3658.6 | 3795.9 | 3712.4 | 3766.7 | 3692.7 | 3736.5 | 3717.3 | 3794.5 | 3755.2 | 3804.7 | 2516.5 |
| Chloroform | 3671.8 | 3658.8 | 3796.3 | 3712.6 | 3766.7 | 3692.8 | 3736.7 | 3717.4 | 3794.8 | 3755.2 | 3804.8 | 2516.3 |
| X2C-TZVPall-s | | | | | | | | | | | | |
| Acetone | 2963.4 | 2947.6 | 3159.1 | 3031.2 | 3095.1 | 2996.3 | 3048.5 | 3031.6 | 3102.3 | 3064.7 | 3119.6 | 2543.1 |
| Dichloromethane | 2963.4 | 2947.6 | 3158.9 | 3031.1 | 3095.0 | 2996.2 | 3048.4 | 3031.5 | 3102.2 | 3064.6 | 3119.6 | 2542.8 |
| Chloroform | 2963.5 | 2947.7 | 3158.6 | 3031.0 | 3094.8 | 2996.1 | 3048.4 | 3031.4 | 3102.2 | 3064.5 | 3119.5 | 2542.7 |

Table S10 – Experimental ^119^Sn NMR chemical shifts and calculated (ppm) according to Equation 1 with different density functionals with DKH2 [SARC-DKH-TZVP(Sn)/DKH-def2-TZVP(other atoms)], ZORA [SARC-ZORA-TZVP(Sn)/ZORA-def2-TZVP(other atoms)], and X2C [X2C-TZVPall-s] methods.

|  | SARC-DKH-TZVP | | | | | | | | | | | | | | |
| --- | --- | --- | --- | --- | --- | --- | --- | --- | --- | --- | --- | --- | --- | --- | --- |
| Structure | | EXP | PBE | BP86 | M06-L | TPSS | r^2^SCAN | B3LYP | PBE0 | CAM-B3LYP | M06-2X | ωB97X-D3 | ωB97M-V | B2PLYP-D3 |  |
| Me_2_Sn(dmit) | | 135.2 | 295.1 | 302.9 | 249.4 | 275.8 | 258.5 | 287.6 | 273.3 | 279.2 | 278.7 | 269.8 | 264.9 | 286.2 |  |
| MePhSn(dmit) | | 75.2 | 235.1 | 240.9 | 192.3 | 216.6 | 203.4 | 232.9 | 220.1 | 229.1 | 227.8 | 220.7 | 216.9 | 184.6 |  |
| Ph_2_Sn(dmit) | | -24.8 | 170.9 | 175.8 | 135.6 | 154.9 | 144.5 | 175.6 | 163.1 | 175.8 | 175.3 | 168.3 | 165.1 | 75.94 |  |
| Et_2_Sn(dmit) | | 165.6 | 316.7 | 324.7 | 265.6 | 295.1 | 278.9 | 311.5 | 295.0 | 304.5 | 302.9 | 293.7 | 288.4 | 279.1 |  |
| Bu_2_Sn(dmit) | | 161.5 | 299.3 | 306.8 | 242.7 | 278.2 | 254.9 | 296.7 | 280.8 | 288.3 | 295.1 | 279.1 | 274.9 | 241.5 |  |
| (C_4_H_7_O_2_)_2_Sn(dmit) | | 84.4 | 252.4 | 259.4 | 198.0 | 227.4 | 212.5 | 251.6 | 236.1 | 245.6 | 255.1 | 236.6 | 229.1 | 170.8 |  |
| (C_5_H_9_O_2_)_2_Sn(dmit) | | 84.2 | 208.5 | 215.0 | 164.8 | 187.1 | 179.6 | 206.0 | 191.5 | 199.4 | 193.9 | 191.8 | 182.6 | 63.75 |  |
| C_26_H_24_SSn | | -115.5 | -18.16 | -16.49 | -27.34 | -27.58 | -20.48 | -6.327 | -11.96 | 0.131 | 7.299 | 1.722 | -2.503 | -145.4 |  |
| C_6_H_12_N_2_S_3_Sn | | -270.1 | -136.5 | -134.1 | -142.5 | -135.0 | -140.0 | -124.0 | -127.1 | -115.4 | -108.9 | -112.9 | -114.2 | -287.4 |  |
| C_16_H_25_N_3_S_3_Sn | | -427.3 | -110.7 | -105.7 | -120.3 | -123.8 | -114.8 | -97.46 | -112.8 | -95.91 | -96.43 | -98.0 | -105.0 | -345.3 |  |
| C_21_H_35_N_3_S_6_Sn | | -813 | -534.5 | -532.0 | -495.3 | -527.6 | -509.7 | -507.9 | -521.6 | -507.9 | -486.0 | -505.0 | -504.2 | -933.3 |  |

Table S10 – Continued

|  | SARC-ZORA-TZVP | | | | | | | | | | | | | |  |
| --- | --- | --- | --- | --- | --- | --- | --- | --- | --- | --- | --- | --- | --- | --- | --- |
| Structure | | EXP | PBE | BP86 | M06-L | TPSS | r^2^SCAN | B3LYP | PBE0 | CAM-B3LYP | M06-2X | ωB97X-D3 | ωB97M-V | B2PLYP-D3 | |
| Me_2_Sn(dmit) | | 135.2 | 288.2 | 295.8 | 243.4 | 269.4 | 252.4 | 280.9 | 267.0 | 272.8 | 272.1 | 263.6 | 258.8 | 279.9 | |
| MePhSn(dmit) | | 75.2 | 230.0 | 235.8 | 188.1 | 212.2 | 199.1 | 228.0 | 215.5 | 224.3 | 222.9 | 216.1 | 212.4 | 180.7 | |
| Ph_2_Sn(dmit) | | -24.8 | 167.8 | 172.7 | 133.2 | 152.3 | 142.1 | 172.6 | 160.4 | 172.7 | 172.1 | 165.4 | 162.4 | 74.50 | |
| Et_2_Sn(dmit) | | 165.6 | 309.4 | 317.3 | 259.4 | 288.5 | 272.6 | 304.4 | 288.4 | 297.7 | 295.9 | 287.1 | 282.0 | 272.8 | |
| Bu_2_Sn(dmit) | | 161.5 | 292.9 | 300.2 | 237.7 | 272.5 | 249.9 | 290.3 | 274.8 | 282.2 | 288.1 | 273.1 | 268.9 | 236.0 | |
| (C_4_H_7_O_2_)_2_Sn(dmit) | | 84.4 | 247.5 | 254.4 | 194.5 | 223.4 | 209.0 | 246.7 | 231.7 | 241.0 | 249.4 | 232.2 | 229.2 | 167.6 | |
| (C_5_H_9_O_2_)_2_Sn(dmit) | | 84.2 | 205.1 | 211.4 | 162.1 | 184.4 | 176.8 | 202.7 | 188.5 | 196.3 | 190.6 | 188.9 | 179.9 | 62.99 | |
| C_26_H_24_SSn | | -115.5 | -17.03 | -15.39 | -26.06 | -26.13 | -19.28 | -5.397 | -10.91 | 0.894 | 7.839 | 2.419 | -1.676 | -142.9 | |
| C_6_H_12_N_2_S_3_Sn | | -270.1 | -133.0 | -130.6 | -139.0 | -131.4 | -136.4 | -120.7 | -123.7 | -112.1 | -105.8 | -109.8 | -110.9 | -281.9 | |
| C_16_H_25_N_3_S_3_Sn | | -427.3 | -106.9 | -102.0 | -116.6 | -119.6 | -110.9 | -93.91 | -108.8 | -92.31 | -93.01 | -94.46 | -101.1 | -337.9 | |
| C_21_H_35_N_3_S_6_Sn | | -813 | -520.5 | -518.2 | -482.7 | -513.7 | -496.4 | -494.5 | -507.9 | -494.4 | -473.2 | -491.6 | -490.7 | -913.8 | |

Table S10 – Continued

|  | X2C-TZVPall-s | | | | | | | | | | | | | |
| --- | --- | --- | --- | --- | --- | --- | --- | --- | --- | --- | --- | --- | --- | --- |
| Structure | | EXP | PBE | BP86 | M06-L | TPSS | r^2^SCAN | B3LYP | PBE0 | CAM-B3LYP | M06-2X | ωB97X-D3 | ωB97M-V | B2PLYP-D3 |
| Me_2_Sn(dmit) | | 135.2 | 44.00 | 53.70 | 12.70 | 28.30 | 16.60 | 37.60 | 26.50 | 34.30 | 30.30 | 28.80 | 26.90 | 304.3 |
| MePhSn(dmit) | | 75.2 | -10.60 | -1.700 | -78.60 | -39.30 | -73.00 | -12.20 | -23.90 | -12.20 | 4.500 | -18.60 | -25.00 | 176.2 |
| Ph_2_Sn(dmit) | | -24.8 | -70.00 | -61.60 | -90.80 | -82.70 | -88.40 | -65.70 | -76.90 | -62.60 | -69.70 | -68.50 | -67.70 | 59.00 |
| Et_2_Sn(dmit) | | 165.6 | 44.50 | 54.50 | -20.60 | 17.10 | -14.10 | 40.40 | 27.80 | 38.10 | 52.70 | 31.40 | 26.40 | 295.5 |
| Bu_2_Sn(dmit) | | 161.5 | 51.60 | 62.40 | -7.100 | 29.80 | 3.500 | 50.20 | 34.90 | 45.60 | 43.20 | 39.30 | 27.80 | 245.5 |
| (C_4_H_7_O_2_)_2_Sn(dmit) | | 84.4 | -19.90 | -10.90 | -80.90 | -45.70 | -66.20 | -20.00 | -33.50 | -21.30 | -22.00 | -28.50 | -38.10 | 175.9 |
| (C_5_H_9_O_2_)_2_Sn(dmit) | | 84.2 | -81.46 | -72.71 | -114.6 | -99.76 | -102.1 | -84.51 | -96.49 | -86.51 | -97.29 | -90.78 | -97.07 | 75.41 |
| C_26_H_24_SSn | | -115.5 | -217.2 | -212.9 | -211.9 | -218.1 | -208.0 | -203.4 | -204.8 | -191.4 | -193.9 | -187.7 | -189.0 | -168.5 |
| C_6_H_12_N_2_S_3_Sn | | -270.1 | -417.2 | -414.4 | -402.0 | -406.8 | -408.2 | -401.7 | -399.7 | -386.2 | -384.2 | -381.6 | -379.5 | -283.9 |
| C_16_H_25_N_3_S_3_Sn | | -427.3 | -480.2 | -473.1 | -476.3 | -486.9 | -474.9 | -465.6 | -478.2 | -458.1 | -468.6 | -459.7 | -462.9 | -355.9 |
| C_21_H_35_N_3_S_6_Sn | | -813 | -863.5 | -859.0 | -794.6 | -846.3 | -818.0 | -823.7 | -833.8 | -813.7 | -795.5 | -812.4 | -808.2 | -965.1 |

Table S11 - Statistical analysis, including 95% confidence intervals and p-values for the regression parameters obtained from the comparison between experimental and calculated ^119^Sn NMR chemical shifts.

| DFT | R² | MD | MAD | RMSD | SD | p-value _Slope_ | Slope | SE* | CI 95% _Slope_ | p-value _Intercept_ | Intercept | SE* | CI 95% _Intercept_ |
| --- | --- | --- | --- | --- | --- | --- | --- | --- | --- | --- | --- | --- | --- |
|  | SARC-DKH-TZVP | | | | | | | | | | | | |
| PBE | 0.968 | 174.78 | 174.78 | 185.86 | 66.31 | 4.74E-08 | 1.142 | 0.069 | [0.99 - 1.3] | 3.09E-06 | -187.4 | 18.42 | [-229.09 - 145.76] |
| BP86 | 0.967 | 180.18 | 180.18 | 190.86 | 66.03 | 5.61E-08 | 1.133 | 0.070 | [0.98 - 1.29] | 3.01E-06 | -192.7 | 18.88 | [-235.45 - 150.03] |
| M06-L | 0.970 | 146.14 | 146.14 | 167.25 | 85.30 | 3.73E-08 | 1.283 | 0.075 | [1.11 - 1.45] | 6.42E-06 | -163.2 | 17.51 | [-202.83 - 123.59] |
| TPSS | 0.971 | 160.52 | 160.52 | 174.00 | 70.42 | 3.05E-08 | 1.186 | 0.068 | [1.03 - 1.34] | 3.35E-06 | -174.4 | 17.30 | [-213.55 - 135.28] |
| r^2^SCAN | 0.970 | 153.81 | 153.81 | 171.36 | 79.21 | 3.53E-08 | 1.242 | 0.072 | [1.08 - 1.41] | 4.52E-06 | -170.3 | 17.52 | [-209.9 - 130.66] |
| B3LYP | 0.968 | 179.16 | 179.16 | 192.05 | 72.57 | 4.73E-08 | 1.188 | 0.072 | [1.03 - 1.35] | 2.25E-06 | -196.7 | 18.61 | [-238.78 - 154.59] |
| PBE0 | 0.972 | 166.47 | 166.47 | 179.88 | 71.48 | 2.60E-08 | 1.198 | 0.067 | [1.05 - 1.35] | 2.12E-06 | -182.4 | 17.13 | [-221.15 - 143.64] |
| CAM-B3LYP | 0.970 | 177.04 | 177.04 | 190.57 | 73.96 | 3.40E-08 | 1.208 | 0.070 | [1.05 - 1.37] | 1.70E-06 | -196.0 | 17.92 | [-236.5 - 155.41] |
| M06-2X | 0.972 | 180.86 | 180.86 | 195.24 | 77.13 | 2.74E-08 | 1.235 | 0.070 | [1.08 - 1.39] | 1.10E-06 | -203.1 | 17.65 | [-243.05 - 163.23] |
| ωB97X-D3 | 0.972 | 171.86 | 171.86 | 186.68 | 76.43 | 2.52E-08 | 1.232 | 0.069 | [1.08 - 1.39] | 1.47E-06 | -191.8 | 17.25 | [-230.84 - 152.78] |
| ωB97M-V | 0.973 | 167.33 | 167.33 | 182.86 | 77.36 | 2.22E-08 | 1.241 | 0.069 | [1.09 - 1.4] | 1.55E-06 | -187.0 | 16.92 | [-225.26 - 148.7] |
| B2PLYP-D3 | 0.975 | 48.64 | 82.83 | 92.68 | 82.74 | 1.54E-08 | 0.818 | 0.043 | [0.72 - 0.92] | 5.82E-03 | -55.40 | 15.42 | [-90.29 - 20.51] |

95% CI calculated using t = 2.262 (df = 9).

*SE = Standard Error.

Table S11 - Continued

| DFT | R² | MD | MAD | RMSD | SD | p-value _Slope_ | Slope | SE* | CI 95% _Slope_ | p-value _Intercept_ | Intercept | SE* | CI 95% _Intercept_ |
| --- | --- | --- | --- | --- | --- | --- | --- | --- | --- | --- | --- | --- | --- |
|  | SARC-ZORA-TZVP | | | | | | | | | | | | |
| PBE | 0.968 | 173.45 | 173.45 | 185.94 | 70.26 | 4.86E-08 | 1.171 | 0.071 | [1.01 - 1.33] | 3.06E-06 | -188.4 | 18.49 | [-230.22 - 146.58] |
| BP86 | 0.967 | 178.73 | 178.73 | 190.73 | 69.83 | 5.75E-08 | 1.161 | 0.072 | [1 - 1.32] | 2.98E-06 | -193.7 | 18.95 | [-236.59 - 150.84] |
| M06-L | 0.970 | 145.34 | 145.34 | 168.69 | 89.83 | 3.78E-08 | 1.315 | 0.077 | [1.14 - 1.49] | 6.26E-06 | -164.0 | 17.55 | [-203.74 - 124.34] |
| TPSS | 0.971 | 159.68 | 159.68 | 174.88 | 74.79 | 3.11E-08 | 1.216 | 0.070 | [1.06 - 1.37] | 3.25E-06 | -175.6 | 17.36 | [-214.86 - 136.32] |
| r^2^SCAN | 0.970 | 153.04 | 153.04 | 172.61 | 83.73 | 3.62E-08 | 1.273 | 0.075 | [1.1 - 1.44] | 4.43E-06 | -171.4 | 17.59 | [-211.17 - 131.61] |
| B3LYP | 0.968 | 177.79 | 177.79 | 192.30 | 76.88 | 4.87E-08 | 1.217 | 0.074 | [1.05 - 1.38] | 2.23E-06 | -197.8 | 18.69 | [-240.06 - 155.5] |
| PBE0 | 0.972 | 165.42 | 165.42 | 180.59 | 75.99 | 2.68E-08 | 1.228 | 0.069 | [1.07 - 1.38] | 2.10E-06 | -183.5 | 17.22 | [-222.46 - 144.58] |
| CAM-B3LYP | 0.970 | 175.79 | 175.79 | 191.05 | 78.48 | 3.49E-08 | 1.238 | 0.072 | [1.07 - 1.4] | 1.67E-06 | -197.2 | 18.00 | [-237.88 - 156.45] |
| M06-2X | 0.972 | 179.23 | 179.23 | 195.48 | 81.82 | 2.77E-08 | 1.266 | 0.072 | [1.1 - 1.43] | 1.08E-06 | -204.1 | 17.69 | [-244.1 - 164.07] |
| ωB97X-D3 | 0.972 | 170.69 | 170.69 | 187.38 | 81.08 | 2.58E-08 | 1.263 | 0.071 | [1.1 - 1.42] | 1.45E-06 | -193.0 | 17.32 | [-232.17 - 153.79] |
| ωB97M-V | 0.973 | 166.72 | 166.72 | 184.13 | 81.97 | 2.36E-08 | 1.271 | 0.071 | [1.11 - 1.43] | 1.55E-06 | -188.6 | 17.07 | [-227.23 - 150.02] |
| B2PLYP-D3 | 0.975 | 49.33 | 78.63 | 88.10 | 76.56 | 1.55E-08 | 0.836 | 0.044 | [0.74 - 0.94] | 5.87E-03 | -55.3 | 15.43 | [-90.21 - 20.43] |

95% CI calculated using t = 2.262 (df = 9).

*SE = Standard Error.

Table S11 – Continued

| DFT | R² | MD | MAD | RMSD | SD | p-value _Slope_ | Slope | SE* | CI 95% _Slope_ | p-value _Intercept_ | Intercept | SE* | CI 95% _Intercept_ |
| --- | --- | --- | --- | --- | --- | --- | --- | --- | --- | --- | --- | --- | --- |
|  | X2C-TZVPall-s | | | | | | | | | | | | |
| PBE | 0.986 | -97.76 | 97.76 | 104.49 | 38.71 | 1.14E-09 | 1.048 | 0.041 | [0.95 - 1.14] | 2.90E-05 | 106.5 | 13.78 | [75.39 - 137.7] |
| BP86 | 0.986 | -90.09 | 90.09 | 97.37 | 38.73 | 1.37E-09 | 1.040 | 0.042 | [0.95 - 1.14] | 6.26E-05 | 97.2 | 13.86 | [65.82 - 128.53] |
| M06-L | 0.982 | -120.01 | 123.35 | 135.64 | 66.31 | 3.25E-09 | 1.208 | 0.054 | [1.09 - 1.33] | 5.11E-06 | 162.9 | 17.01 | [124.43 - 201.36] |
| TPSS | 0.987 | -109.61 | 109.61 | 117.52 | 44.46 | 8.24E-10 | 1.099 | 0.042 | [1 - 1.19] | 6.17E-06 | 129.1 | 13.78 | [97.88 - 160.24] |
| r^2^SCAN | 0.984 | -117.11 | 117.11 | 129.56 | 58.12 | 1.97E-09 | 1.167 | 0.049 | [1.06 - 1.28] | 5.08E-06 | 151.1 | 15.76 | [115.43 - 186.71] |
| B3LYP | 0.985 | -91.26 | 91.26 | 101.29 | 46.10 | 1.56E-09 | 1.097 | 0.045 | [1 - 1.2] | 3.41E-05 | 108.4 | 14.31 | [76.07 - 140.83] |
| PBE0 | 0.987 | -101.23 | 101.23 | 110.25 | 45.81 | 9.65E-10 | 1.105 | 0.043 | [1.01 - 1.2] | 1.09E-05 | 120.9 | 13.84 | [89.59 - 152.21] |
| CAM-B3LYP | 0.986 | -88.11 | 88.11 | 99.74 | 49.00 | 1.37E-09 | 1.118 | 0.045 | [1.02 - 1.22] | 2.99E-05 | 108.7 | 14.12 | [76.8 - 140.66] |
| M06-2X | 0.984 | -86.89 | 90.07 | 100.08 | 52.09 | 2.46E-09 | 1.126 | 0.049 | [1.02 - 1.24] | 5.01E-05 | 108.7 | 15.07 | [74.62 - 142.79] |
| ωB97X-D3 | 0.986 | -91.25 | 91.36 | 103.15 | 50.44 | 1.06E-09 | 1.132 | 0.044 | [1.03 - 1.23] | 1.69E-05 | 114.6 | 13.85 | [83.26 - 145.92] |
| ωB97M-V | 0.985 | -94.72 | 95.59 | 107.63 | 53.60 | 1.45E-09 | 1.145 | 0.047 | [1.04 - 1.25] | 1.57E-05 | 121.0 | 14.49 | [88.18 - 153.73] |
| B2PLYP-D3 | 0.975 | 45.73 | 87.12 | 99.73 | 92.95 | 1.62E-08 | 0.793 | 0.042 | [0.7 - 0.89] | 6.93E-03 | -54.1 | 15.52 | [-89.16 - 18.96] |

95% CI calculated using t = 2.262 (df = 9).

*SE = Standard Error.

**References**

1.	Munguia T, Pavel IS, Kapoor RN, Cervantes-Lee F, Párkányi L, Pannell KH (2003) Lewis acidity of group 14 elements toward intramolecular sulfur in ortho-aryl-thioanisoles. Can J Chem 81:1388–1397. https://doi.org/10.1139/v03-163

2.	Chiorean N, Coza C, Pop A, Silvestru A (2019) New hypercoordinated diorganotin(IV) compounds with dithiocarbamato ligands. Synthesis and structural characterization. J Organomet Chem 880:83–90. https://doi.org/10.1016/j.jorganchem.2018.10.030

3.	Dakternieks D, Zhu H, Masi D, Mealli C (1993) Stereochemistry of hypervalent tin (IV) compounds. NMR and crystallographic studies of organoyltin (IV) complexes with 1, 1-dithiolate ligands. Inorganica Chim Acta 211:155–160. https://doi.org/10.1016/S0020-1693(00)85595-4

4.	Doidge-Harrison SMS V, Irvine JTS, Khan A, Spencer GM, Wardell JL, Aupers JH (1996) Diorganotin 1,3-dithiole-2-thione-4,5-dithiolate compounds, R 2Sn(dmit): the crystal structure of MePhSn(dmit). J Organomet Chem 516:199–205. https://doi.org/10.1016/0022-328X(96)06139-6

5.	Buchanan H, Howie RA, Khan A, Spencer GM, Wardell JL, Aupers JH (1996) Preparations and crystal structures of Sn(CH2CH2C02Me),(C3SS) and [ Q] [ Sn(CH2CH2C02Me)(C3SS)),] (Q = NEt, or 1,4-dimethylpyridinium, C,SS = 4,5=disulfanyl=l,3dithiole-2=thionate). Journal of the Chemical Society, Dalton Transactions 541–548. https://doi.org/10.1039/DT9960000541

6.	Lima GMD, Wardell SMSV, Wardell JL, Tiekink ERT (2009) Bis(2-ethoxy-carbonyl-ethyl-κ2 C 1,O)(2-thioxo- 1,3-dithiole-4,5-dithio-ato-κ2 S 4,S 5)tin(IV). Acta Crystallogr Sect E Struct Rep Online 65: m1635–m1636. https://doi.org/10.1107/S1600536809048971

7.	Comerlato NM, Ferreira GB, Howie RA, Silva CXA, Wardell JL (2008) Synthesis of bis(tert-butyl)(2-thioxo-1,3-dithiole-4,5-dithiolato) stannane, But_2Sn(dmit), and bis(tert-butyl)(2-oxo-1,3-dithiole-4,5-dithiolato)stannane, But_2Sn(dmio): Crystal structures, at 120 K, of polymeric But_2Sn(dmit), and two polymorphs of But_2Sn(dmio), a polymer and a cyclic tetramer. J Organomet Chem 693:2424–2430. https://doi.org/10.1016/j.jorganchem.2008.04.019

8.	Howie RA, Wardell JL (2001) Dichlorobis (4-methylpentan-2-onato-C4, O) tin (IV) and bis (4-methylpentan-2-onato-C4, O)(2-thioxo-1, 3-dithiole-4, 5-dithiolato-S, S’) tin (IV) at 150 K. Crystal Structure Communications 57:1041–1043. https://doi.org/10.1107/S0108270101009659

9.	Howie RA, de Lima GM, Wardell JL, Wardell SMS V, Comerlato NM (2011) Bis(2-amidoethyl)tin compounds, (H_2NCOCH_2CH_2-C,O)_2SnX_2, and complexes: supramolecular arrays derived from hydrogen bonds involving the amido and X units. Z Kristallogr Cryst Mater 226:535–552. https://doi.org/10.1524/zkri.2011.1369

10.	Allan GM, Howie RA, Skakle JMS, Wardell JL, Wardell SMS V (2001) Further studies on dialkyltin 1, 3-dithiole-2-thione-4, 5-dithiolates, R2Sn (dmit). Crystal structures of orthorhombic-and monoclinic-Et2Sn (dmit), and Me2Sn (dmit). J Organomet Chem 627:189–200. https://doi.org/10.1016/S0022-328X(01)00735-5

11.	Skakle JMS, Wardell JL, Wardell SMS V (2001) Diphenyl (2-thioxo-1, 3-dithiole-4, 5-dithiolato-S, S’) plumbane at 295 K and diphenyl (2-thioxo-1, 3-dithiole-4, 5-dithiolato-S, S’) stannane at 150 K. Crystal Structure Communications 57:787–789. https://doi.org/10.1107/S0108270101004474
